# Targeting ferroptosis with the lipoxygenase inhibitor PTC-041 as a therapeutic strategy for the treatment of Parkinson’s disease

**DOI:** 10.1371/journal.pone.0309893

**Published:** 2024-09-18

**Authors:** Angela Minnella, Kevin P. McCusker, Akiko Amagata, Beatrice Trias, Marla Weetall, Joey C. Latham, Sloane O’Neill, Richard K. Wyse, Matthew B. Klein, Jeffrey K. Trimmer

**Affiliations:** 1 PTC Therapeutics, Mountain View, California, United States of America; 2 PTC Therapeutics, Warren, New Jersey, United States of America; 3 Cure Parkinson’s, London, England, United Kingdom; Toho University Graduate School of Medicine, JAPAN

## Abstract

Parkinson’s disease is the second most common neurodegenerative disorder, affecting nearly 10 million people worldwide. Ferroptosis, a recently identified form of regulated cell death characterized by 15-lipoxygenase-mediated hydroperoxidation of membrane lipids, has been implicated in neurodegenerative disorders including amyotrophic lateral sclerosis and Parkinson’s disease. Pharmacological inhibition of 15 -lipoxygenase to prevent iron- and lipid peroxidation-associated ferroptotic cell death is a rational strategy for the treatment of Parkinson’s disease. We report here the characterization of PTC-041 as an anti-ferroptotic reductive lipoxygenase inhibitor developed for the treatment of Parkinson’s disease. In these studies, PTC-041 potently protects primary human Parkinson’s disease patient-derived fibroblasts from lipid peroxidation and subsequent ferroptotic cell death and prevents ferroptosis-related neuronal loss and astrogliosis in primary rat neuronal cultures. Additionally, PTC-041 prevents ferroptotic-mediated α-synuclein protein aggregation and nitrosylation in vitro, suggesting a potential role for anti-ferroptotic lipoxygenase inhibitors in mitigating pathogenic aspects of synucleinopathies such as Parkinson’s disease. We further found that PTC-041 protects against synucleinopathy in vivo, demonstrating that PTC-041 treatment of Line 61 transgenic mice protects against α-synuclein aggregation and phosphorylation as well as prevents associated neuronal and non-neuronal cell death. Finally, we show that. PTC-041 protects against 6-hydroxydopamine-induced motor deficits in a hemiparkinsonian rat model, further validating the potential therapeutic benefits of lipoxygenase inhibitors in the treatment of Parkinson’s disease.

## Introduction

Parkinson’s disease (PD) is a devastating, progressive neurodegenerative disorder that has doubled in prevalence within the last 30 years, affecting nearly 10 million people worldwide [1]. The number of patients diagnosed with PD increases significantly over the age of 60 [2]. As the elderly population continues to expand globally, a sharp rise in the prevalence of PD is predicted by 2040, with the number of individuals with PD anticipated to exceed 12 million worldwide [3]. Current therapies for PD are moderately effective in symptom management; however, due to the progressive nature of the disease, patients may experience waning of drug benefits and increased unwanted side effects with debilitating clinical consequences as they advance to later stages in PD pathology. There remains an urgent, unmet need for the development of novel, disease-modifying therapies to slow or halt disease progression [4, 5]. However, the biological mechanisms underpinning PD pathology are not well characterized, which impedes this quest for new treatment paradigms. Because PD pathogenesis begins long before the manifestation of clinical symptoms, further characterization, and subsequent targeting of early pathophysiologic processes in PD may prevent or delay clinical onset of the disease.

**Fig 1:**
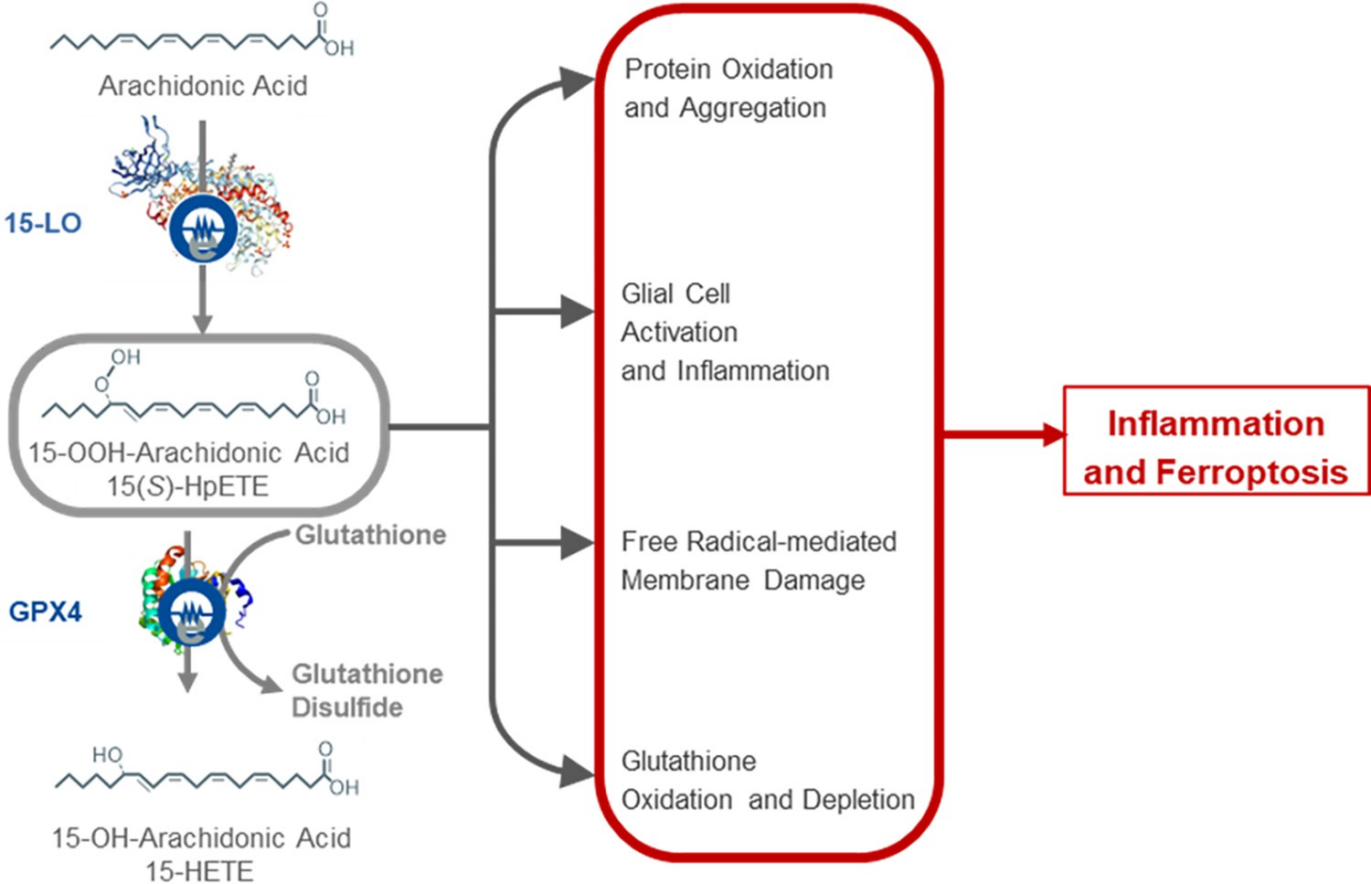
The role of lipoxygenase enzymes as key regulators in ferroptotic cell death. Lipoxygenase (LO) enzymes oxidize free- and membrane-bound polyunsaturated fatty acids into an array of lipid species, which are further enzymatically transformed into many lipid signaling molecules [15]. Leukotrienes, prostaglandins, hydroxyeicosatetraenoic acids (HETEs), and thromboxanes are all formed from arachidonic acid. Hydroperoxyeicosatetraenoic acids (HpETEs), the precursors of HETEs, are critically involved in ferroptotic cell death [6–8]. Increased 15-LO activity leads to glutathione oxidation and depletion, neuroinflammation, protein aggregation, and, ultimately, ferroptotic cell death.

Recent studies have elucidated the growing importance of iron metabolism in the early pathophysiologic processes associated with a variety of neurodegenerative disorders, including amyotrophic lateral sclerosis and PD. Specifically, the role of ferroptosis, an iron-dependent, non-apoptotic cell death pathway characterized by reduced intracellular glutathione levels, increased reactive oxygen species (ROS), and elevated lipid peroxidation, is gaining prominence as one of the key pathological mechanisms underlying neurodegeneration (Fig 1) [6–11]. In addition to the accumulation of iron-dependent free radicals, it has been demonstrated that the peroxidation of polyunsaturated fatty acids (PUFAs), a process mediated by lipoxygenase (LO) enzymes within the ferroptotic pathway, underlies α-synuclein protein aggregation, which is a key pathological hallmark of PD [12, 13]. The link between ferroptosis and PD in dopaminergic neurons of the substantia nigra of PD patients has recently been further highlighted at the single-cell transcriptomic level [14].

15-Lipoxygenase (15-LO) is an oxidoreductase that catalyzes the transfer of electrons to membrane-associated PUFAs, resulting in the generation of lipid hydroperoxide signals that induce ferroptosis and inflammation [8, 16]. Several pathological and biochemical events associated with 15-LO activation are observed in the substantia nigra (SN) of patients with PD, including (i) iron accumulation [17, 18]; (ii) glutathione depletion resulting from chronic oxidative stress [19, 20]; (iii) elevated levels of lipid peroxidation products [21, 22]; and (iv) activation of microglial cells and astrocytes [23, 24]. Other biochemical markers of lipid peroxidation have also been detected in the SN of patients with PD, such as elevated levels of a lipid hydroperoxidation end-product, 4-hydroxynonenal, a PUFA metabolite [25]. Additionally, plasma levels of malondialdehyde (MDA) correlate with PD staging, indicating that plasma MDA levels may function as a sensitive biomarker for the early detection of PD [22]. Overall, the accumulated evidence connecting ongoing oxidative stress, lipid peroxidation and ferroptosis with LO enzyme activity to the neurodegeneration observed in patients with PD supports further investigation of drugs that target this specific pathway. Data from in vitro models [26] further support that anti-ferroptotic treatment for PD is a promising therapeutic path. Taken together, these findings provide a strong rationale for developing LO inhibitors as a neuroprotective, disease-modifying therapeutic strategy for the treatment of PD.

Here, we provide preclinical proof-of-concept that the anti-ferroptotic 15-LO inhibitor, PTC-041, could be beneficial for use in the treatment of PD. We characterized its LO inhibitory activity in vitro, using PD patient-derived fibroblasts, and further demonstrated its neuroprotective properties against neurite degeneration and astrogliosis in neuronal co-cultures from embryonic rat midbrains. We then validated these findings using in vivo animal models of PD, demonstrating not only protection against functional impairments associated with dopaminergic neuronal cell death in the SN but also a reduction in pathological α-synuclein aggregation in the brains of treated Line 61 mice.

## Materials and methods

### In vitro methods

#### Lipoxygenase assay

All enzymatic activity was assessed by product formation using liquid chromatography with tandem mass spectrometry (LC-MS/MS) to separate and quantify the various fatty acyl species present. For half-maximal inhibitory concentration (IC_50_) determinations, varying concentrations of the active hydroquinone form of PTC-041 were prepared in dimethylsulfoxide (DMSO) in an anerobic glovebox and were removed immediately before dosing to reactions.

Lysates containing active 5-LO were prepared from Expi293F cells transfected with 5-LO-bearing plasmid. Ten million cells were resuspended in 0.2 mL lysis buffer (20 mM tris-hydrogen chloride [HCl], pH 7.5, containing 5% volume/volume [v/v] glycerol and halt protease inhibitor) and lysed by sonication with a tip sonicator (3 bursts of 1 second each, with 5 seconds in between, were used at 20% attenuation). Cell debris was removed by centrifugation, and supernatant was used as-is for subsequent experiments.

Enzyme concentrations were as follows: 20 nM 12-LO or 15-LO; 5-LO-overexpressing cells were lysed as indicated above, and the resulting 5-LO-containing supernatant was diluted 20-fold into the final assay mixture. Assays for all LO isoforms evaluated were performed in 20 mM tris-HCl pH 7.5, 5% residual DMSO from inhibitor or vehicle additions. Arachidonic acid (AA) was varied in an enzyme- and assay-specific manner, but for standard IC_50_ determinations, the AA concentrations were as follows: 10 μM for 15-LO and 12-LO; 20 μM for 5-LO.

All assays were run on the Tecan Fluent to facilitate liquid handling under kinetic conditions: reactions were initiated by the addition of AA and were quenched at appropriate time intervals into 4 volumes of methanol containing tris(2 carboxyethyl)phosphine and internal standard to halt the reaction and convert HpETE/epoxy products into the corresponding stable HETEs. Reaction products were separated by ultra performance liquid chromatography, and concentrations determined by mass spectroscopy, using a standard curve of the authentic standards. Enzymatic rates were determined by least-squares linear fits of [P] versus time plots, and normalized activities (vehicle only = 100%) were fitted by plotting activity versus log [compound] using a standard 4-parameter logistic fit.

#### Expi293F cell culture and transfection

Cells were obtained from ThermoFisher (Cat# AK1527, October 2020) and cultured in Expi293 Expression Medium without complementation. For routine culture, cells were passaged every 3 to 4 days, maintaining a density below 6 × 10^6^ cells/mL culture. Cells were passaged at least 3 times post-thaw prior to transfection. Transfection with empty plasmid, control plasmid containing green fluorescent protein (GFP), and plasmid bearing full-length human 5-LO was accomplished using ExpiFectamine 293 reagents and Opti-MEM I media as per the manufacturer’s instructions. Transfected cells were sub-cultured for 48 to 72 hours and frozen in single-use aliquots of 10 or 20 million cells suitable for lysis and used in activity assays.

#### Ferroptosis-induced cell death

Ferroptotic cell death was induced in the mouse striatal cell line, Q7 cells using the glutathione peroxidase-4 (GPX4) inhibitor, RSL3. Q7 cells were seeded in assay medium (DMEM + 10% FBS + penicillin/streptomycin) and seeded at 500 cells per well in clear-bottom, black-wall, 384-well, tissue culture-treated polystyrene microplates (Corning) using either an electronic multichannel pipette or a Multidrop™ Combi Reagent Dispenser (ThermoFisher Scientific). Cells were incubated for 5 hours at 33°C (95% humidity, 5% CO_2_) to allow attachment. A D300e Digital Dispenser (Tecan) was used to administer test compounds to the desired final concentrations, followed within 15 minutes by RSL3 (2 μM final concentration). DMSO diluent was backfilled to a final concentration of 0.3% (v/v). Cell plates were incubated for 18 hours at 33°C (95% humidity, 5% CO_2_). After a 15-minute equilibration to room temperature, cell viability was assessed using CellTiter-Glo^®^ 2.0 (Promega), which was added using the Multidrop™ Combi Reagent Dispenser (ThermoFisher). After 15 minutes of incubation at room temperature in the dark, the luminescence per well was determined by the Infinite M1000 plate reader (Tecan, 100 ms integration time). Data were analyzed using GraphPad Prism software. half-maximal effective concentration (EC_50_) values were estimated using standard four-parameter curve fitting algorithms.

#### Caspase activity assay

Apoptosis was induced in Q7 cells using staurosporine. Q7 cells were seeded in assay medium (DMEM + 10% FBS + penicillin/streptomycin) and seeded at 3000 cells per well in white-wall, 384-well, tissue culture-treated polystyrene microplates (Corning) using either an electronic multichannel pipette or a Multidrop™ Combi Reagent Dispenser (ThermoFisher). Cells were incubated for 18 hours at 33°C (95% humidity, 5% CO_2_) to allow attachment. A D300e Digital Dispenser (Tecan) was used to administer test compounds to the desired final concentrations, followed within 15 minutes by staurosporine (1 μM final concentration). DMSO diluent was back-filled to a final concentration of 0.3% (v/v). Cell plates were incubated for 6 hours at 33°C (95% humidity, 5% CO_2_). After a 30-minute equilibration to room temperature, Caspase 3/7 activity was assessed using Caspase-Glo^®^ 3/7 assay system (Promega), which was added using an electronic multichannel pipette. After 15 minutes of incubation at room temperature in the dark, the luminescence per well was determined by the Infinite M1000 plate reader (Tecan, 100 ms integration time). Data were analyzed using GraphPad Prism software.

#### Primary patient fibroblast culture

Cells were obtained from Rutgers (see Table 3 for details) between August 2017 –June 2018. They were cultured in Dulbecco’s Modified Eagle Medium (Gibco) supplemented with fetal bovine serum (10% v/v; Sigma), penicillin (100 U/mL; Gibco), and streptomycin (100 μg/mL; Gibco). For routine culture, fibroblast cells were passaged every 4 to 7 days, always maintaining sub-confluent cell densities. Culture medium was exchanged every 3 to 4 days. All experiments described herein were performed within 7 passages of initial cryorecovery.

#### Cell survival assay and cell lipid oxidation assay

Ferroptotic cell death was induced in patient-derived fibroblasts using the GPX4 inhibitor RSL3 (2 μM) [27]; cell survival and lipid oxidation were assayed as previously described [28, 29]. Complete viability (100%) was defined as the average value for control wells in which RSL3 and vitamin K2 (3 μM) were added. This was comparable to the viability observed in wells in which no RSL3 was added. A 0% viability was defined as the average signal observed in control wells in which only RSL3 was added. EC_50_ values were estimated using standard 4-parameter curve fitting algorithms.

Cellular lipids were analyzed using targeted LC-MS/MS as described by Kahn-Kirby et al. [29].

#### Cell viability assay using PD patient-derived fibroblasts

Fibroblasts from donor cells (see Table 3 for details) were detached by trypsinization, resuspended in culture medium, and seeded at 500 cells per well in the wells of 384-well tissue culture-treated polystyrene microplates (clear bottom, black wall; Corning) using the Multidrop™ Combi Reagent Dispenser (ThermoFisher). Cell plates were incubated for 18 hours at 37°C (95% humidity, 5% CO_2_) to allow cell attachment. Test compound (PTC-041) was added to cells using D300e Digital Dispense (Tecan). Cell plates were incubated for 96 hours at 37°C (95% humidity, 5% CO_2_). After a 30-minute equilibration to room temperature, cell viability was assessed by CellTiter-Glo^®^ 2.0 (Promega), which was dispensed to the wells using the Multidrop Combi Reagent Dispenser (ThermoFisher). After 15 minutes of incubation at room temperature in the dark, the luminescence per well was determined on a Synergy plate reader (BioTek, 100 ms integration time). Luminescence values for each well were expressed as a percent of the average of the DMSO-only control well group (defined as 100%). Normalized data were analyzed using GraphPad Prism software, with IC_50_ values estimated using standard 4-parameter curve fitting algorithms.

#### Primary neuronal cultures

Mixed neuron/astrocyte co-cultures were prepared as previously described [30] from the midbrain of embryonic Day 18 Sprague-Dawley rats and plated in 96-well black-walled culture plates (cat. #353219, BD Falcon) coated with laminin and poly-D-lysine. Cells were plated at a density of 12000 cells/100 μL and were subsequently maintained in Neurobasal-A medium (Gibco) containing 1% fetal bovine serum, 5 mM glucose (Gibco), 230 μM sodium pyruvate (Gibco), 1 mM L-glutamine (Gibco), 2% B27 supplement (Gibco), and 0.5% penicillin/streptomycin (Gibco).

#### Lipoxygenase gene expression analysis in primary neuronal cultures

Total RNA from roughly 600,000 cultured primary neurons at DIV1 after seeding was extracted using the NucleoSpin RNA Plus kit (Macherey-Nagel). Complementary deoxyribonucleic acid (cDNA) was prepared using 1 μg of total RNA with the ABI High-Capacity cDNA Reverse Transcription Kit at 60°C (ThermoFisher). cDNA was diluted 5-fold and was used in downstream quantitative polymerase chain reactions with PerfeCTa FastMix II (Quanta) and TaqMan probes (ThermoFisher) on a LightCycler480 instrument (Roche). *Rplp2* (Rn01479927_g1) was used as the normalizing gene. TaqMan assays included *Alox12* (Rn01461081_m1), *Alox12b* (Rn01751249_m1), *Alox15* (Rn01646191_m1), *Alox15b* (Rn00596246_m1), and *Alox5* (Rn00563172_m1). Reactions were run in technical triplicate. Data were analyzed in Excel using the Δ cycle threshold (ΔCt) method.

#### Neurite integrity and astrocyte activation assays

Ferroptotic cell death was induced in primary rat midbrain neuronal cultures 24 hours after plating (DIV1) with the GPX4 inhibitor RSL3 (1.25 μM) [27]. Immediately prior to compound addition, culture media was exchanged for Neurobasal-A medium (without B27 supplement). RSL3 and PTC-041 compound addition was performed with a D300e Digital Dispenser (Tecan). After compound addition, neurite length was monitored in real time with an IncuCyte S3 Live-Cell analysis system (Sartorius). Images were collected every 2 hours for the duration of the experiment. Neurite length was calculated at each timepoint, using the IncuCyte S3 software (NeuroTrack Analysis ID 1283; Cell-Body Cluster Segmentation Adjustment: 0.4, Minimum Cell Width 7 μm; Neurite Parameters: Neurite Sensitivity 0.25, Neurite Width 2 μm), and each well’s value expressed relative to the neurite length of that well at the beginning of the experiment (time 0). Then, 24 hours after the onset of RSL3-mediated neurite injury, the activity and potency of PTC-041 was determined using standard 4-parameter curve fitting using GraphPad Prism software. To quantify astrocyte morphology, cells were washed with phosphate buffered saline (PBS) and fixed with 4% paraformaldehyde, then processed using standard immunocytochemistry techniques. Cells were blocked for 30 minutes (PBS with 2% normal goat serum, 0.2% TritonX-100, and 0.1% bovine serum albumin) and then incubated with primary antibodies to glial fibrillary acidic protein (GFAP; Cell Signaling, 12389, rabbit mAB, 1:250). The primary antibody was recognized by incubating cells with a fluorescently conjugated secondary antibody (Invitrogen, Alexa488-conjugated goat anti-rabbit, A32731). Astrocyte morphology was quantified with the ArrayScan XTI high-content imaging platform (ThermoFisher), focusing on decreased GFAP+ Process Length (HSC Navigator Cellomics, Neuronal Profiling V4.2 BioApplication) as a surrogate for astrocyte activation by analyzing 9 fields per well and 6 wells per condition to yield a total of N>10000 cells analyzed per experimental condition.

Neurite integrity was also quantified in unchallenged neurons that were treated with PTC-041 alone in dose response. For this study, primary rat midbrain neuronal cultures were established as described above, then matured to DIV11 in standard Neurobasal-A medium. Neurite integrity was assayed after 24 hours of PTC-041 treatment, using the IncuCyte S3 Live-Cell analysis system as described above.

#### Generation of N27 cells stably expressing human α-synuclein

The N27 rat dopaminergic neural cell line (Millipore Sigma, Cat# SCC048, January 2019) was transiently transfected with GFP-tagged human α-synuclein (Origene) using Lipofectamine 3000 (Life Technologies). Transfected cells were then selected using Geneticin (G418, 500 μM) until resistant colonies expanded. Stable expression of human α-synuclein gene and protein expression were confirmed via immunocytochemistry, live fluorescence microscopy, and flow cytometry. Cells were maintained in selection media consisting of RPMI-1640 medium supplemented with 10% fetal bovine serum (Sigma), 1% penicillin/streptomycin (Gibco), 2 mM L-glutamine (Gibco), and 500 μM Geneticin. For functional assays, cells were plated in 96well black-walled culture plates (Corning) at 3000 cells/100 μL per well.

#### Cell viability assay using N27 cells

A 40 nM solution of GPX4 inhibitor, RSL3, was added to N27 cells that stably expressed α-synuclein to induce ferroptotic cell death. RSL3 and PTC-041 were added to cells using a D300e Digital Dispenser (Tecan). Cell viability was quantified by the addition of Cytotox Red, a fluorescent indicator that selectively labels dead cells (Sartorius, 4632; excitation/emission 612 nm/631 nm). Immediately prior to adding RSL3 and PTC-041, Cytotox Red (250 nM) was added directly to the media, and cell death was monitored in real time with an IncuCyte S3 Live-Cell analysis system (Sartorius). Images were collected every 2 hours for the duration of the experiment, and cell death was quantified using the IncuCyte S3 software (Basic Analyzer: Analysis 1096, Segmentation: Top-Hat 100 μm radius, Threshold 2.0 RCU, Filters: Minimum Area 75 μm^2^). Values are reported as the average Red Object Count per well, with 4 fields per well and 4 wells per condition analyzed. Data were analyzed using GraphPad Prism software, with IC_50_ values estimated using standard 4-parameter curve fitting algorithms.

#### α-Synuclein immunocytochemistry assays

To quantify α-synuclein protein state in N27 cells, 24 hours after RSL3 and PTC-041 addition, cells were washed with PBS and fixed with 4% paraformaldehyde. Standard immunocytochemistry techniques were used to detect both total and nitrated α-synuclein. Cells were blocked for 30 minutes (PBS with 2% normal goat serum, 0.2% Triton X-100, and 0.1% bovine serum albumin) and then incubated with primary antibodies to total α-synuclein (Abcam, Ab15530, rabbit pAb, 1:50) or nitrated α-Synuclein (Invitrogen, MA5-16142, mouse mAb, 1:250). The primary antibodies were recognized by incubating cells with fluorescently conjugated secondary antibodies (Invitrogen). To enable the quantification of aggregated α-synuclein, monomeric, non-aggregated α-synuclein was extracted by treating cells with 1% Triton X-100 for 48 hours at room temperature [31]. Stained cells were imaged and quantified using the ArrayScan XTI high-content imaging platform (ThermoFisher), focusing on total fluorescence-positive intensity within cells (HSC Navigator Cellomics, Compartmental Analysis V4. 10X, 3Ch, BioApplication) by analyzing >9 fields per well and 6 wells per condition to yield a total of N>10000 cells analyzed per condition.

### In vivo methods

All animal studies were approved by an Institute Animal Care and Use Committee and done in an AAALAC-certified facility.

#### 6-OHDA modeling of PD

Adult female Sprague Dawley rats (175-200g) were purchased from Charles River Laboratories and group housed under environmentally controlled conditions (lights on at 7:00 a.m. and lights off at 7:00 p.m.; 24 ± 2°C constant room temperature). Animals were allowed to acclimate for one week after their arrival and had free access to chow and water. Animal experiments were conducted under the animal protocol reviewed and approved by the Rutgers Institute Animal Care Use Committee (Protocol number 202000063). The ARRIVE guidelines were used to design the experiments. All surgery was performed under isoflurane anesthesia, and meloxicam was administered post-surgery for post-operative pain control. To simulate PD in vivo, rats were unilaterally injected with the dopamine analog 6-OHDA into the medial forebrain bundle (MFB). Sham animals received identical unilateral injections of vehicle (sterile 0.9% saline) into the MFB. A total of 48 female WT Sprague-Dawley rats (Charles River Labor) were randomized into 4 groups (12 rats/group) and injected with 4 μL of either vehicle (Group 1) or 14 μg of 6-OHDA (Groups 2 to 4) unilaterally via intracranial injection into the right MFB. Twenty-four hours post-lesion, Groups 1 and 2 received only sesame oil (5 mL/kg body weight), while Group 3 received a single oral dose of PTC-041 (5 mL/kg body weight). Group 4 was treated with a single dose of PTC-041 24 hours before the lesion. All groups were treated daily with either sesame oil or PTC-041 for a total of 16 days. The rats were weighed before dosing and weekly after they recovered. The study design is described in Table 1.

**Table 1:** Study design and group designation.

Group	Treatment	Test compound	Initiation of dose	Compound dose (mg/kg)	ROA	Number of rats
1	Vehicle	Sesame oil	24h post-lesion	-	PO	12
2	6-OHDA	Sesame oil	24h post-lesion	-	PO	12
3	6-OHDA	PTC-041	24h post-lesion	300	PO	12
4	6-OHDA	PTC-041	24h pre-lesion	300	PO	13

All animals were orally administered PTC-041 or sesame oil daily for 16 days. PTC-041 was administered at 300 mg/kg, a dosing volume of 5 mL/kg body weight, and dosing solution concentration of 60 mg/mL.

**Abbreviations:** 6-OHDA, 6-hydroxydopamine; PO, per os (orally); ROA, route of administration

Apomorphine-induced rotation and cylinder tests were conducted at 14 and 15 days post-lesion, respectively, to evaluate the Parkinsonian phenotype and determine the in vivo efficacy of PTC-041 in 6-OHDA-lesioned rats [32, 33].

*Cylinder test*. A cylinder test was conducted at 2 weeks post-lesion to evaluate the rearing behavior and spontaneous activity of rats. Forelimb use asymmetry was evaluated by measuring weight bearing contacts on the cylinder wall with both the contralateral (left) and ipsilateral (right) paws. Rats were placed in clear acrylic cylinders on top of a clear plastic box that contained a handheld mirror, and the reflection was recorded. The contralateral paw was marked with a Sharpie to assist the scorer. The rats were acclimated to the room for 30 minutes prior to the test. Each test was 10 minutes in length. The total number of touches with the right, left, and both forepaws was counted by a blinded-independent scorer. The results of the test were quantified as a percentage of forepaw preference.

*Apomorphine-induced rotation test*. Following injection with apomorphine (0.5 mg/kg), rats were placed in opaque acrylic bowls and recorded for 40 minutes with the camera mounted to the ceiling. The rats were acclimated to the room for 30 minutes prior to the test. A cue was placed in the 12 o’clock position on the bowl and was used as a starting point for the scorer. When scoring, the head was observed, and the total number of contralateral (counterclockwise) and ipsilateral (clockwise) rotations was counted. One full rotation was defined as 1 complete 360-degree rotation from the nose-point to the cue. The net contralateral rotations were divided by the length of the test (40 minutes) and quantified as turns per minute.

#### Line 61 mouse model of PD

Studies utilizing the Line 61 mouse model of Parkinson’s disease were done by QPS Neuropharmacology (Grambach, Austria). A total of 40 transgenic male Line 61 mice (~6 weeks of age; allocated to 4 groups with n = 10 animals each) and 2 groups with n = 10 animals of non-transgenic age-matched littermates were used for the experiment. Animals received compound (PTC-041, 300 mg/kg) or vehicle once daily for 6 or 15 weeks via oral gavage. To achieve proper compound exposure, animals were dosed in a fed state. For imaging-based readouts, 4 mice per group were sacrificed by cervical dislocation. Brains were carefully removed and washed with ice-cold PBS (pH 7.4). Once rinsed, brains were hemisected and flash frozen in a dry ice/isopropanol bath. Hemibrains were stored at -80⁰C in separate pre-weighed tubes, making sure not to disturb tissue morphology. For the immunofluorescence labeling experiment, a uniform systematic random set of 5 sections per mouse was selected (1 section each from levels 2, 4, 6, 8, and 10). The following multichannel immunofluorescence labeling experiment was executed using rat anti-human α-synuclein monoclonal antibody [15G7] (Enzo Life Sciences, ALX-804-258) and rabbit anti-pSer129- α-synuclein monoclonal antibody [EP1536Y] (Abcam, ab51253; 1/2000 dilution). All sections were counterstained with the nuclear dye 4’,6-diamidino-2-phenylindole (DAPI). Binding of primary antibodies was visualized using highly cross-absorbed secondary antibodies. Whole-slide scans of the stained sections were recorded on a Zeiss automatic microscope AxioScan Z1 with high-aperture lenses, equipped with a Zeiss Axiocam 506 mono and a Hitachi 3CCD HV-F202SCL camera and Zeiss ZEN 2.3 software.

For biochemical determinations of α-synuclein readouts, the left cortex of 6 animals per group was homogenized in 1x lysis buffer (20 mM Tris-HCl, pH 7.4, 500 mM NaCl, 1% Triton X-100), 0.2 mM sodium orthovanadate protease inhibitor cocktail (Calbiochem) and phosphatase inhibitor cocktail (Millipore Sigma) with a UPHO beadmill and metallic beads 3 mm (Biowire) for 60 seconds at 60 Hz. The cortex was homogenized in 9 volumes of buffer. Homogenates were incubated for 30 minutes on ice, followed by centrifugation at 15,000xg for 60 minutes at 4°C. The supernatant was collected as the Triton X-100 soluble fraction. The Triton X-100-insoluble pellet was washed once in lysis buffer and then dissolved in the lysis buffer containing 2% SDS. The resulting homogenate in 2% SDS was collected at the Triton X-100 insoluble fraction. Total protein levels of all samples were determined using Pierce^™^ BCA assay (Thermo Fisher Scientific, 23225). Human total α-synuclein levels were analyzed in both fractions.

*Human total α-synuclein Meso scale discovery assay*. Human α-synuclein levels in the Triton X-100 soluble and insoluble fractions of all samples (n = 240) were determined in duplicates by using a commercially available immunosorbent assay (Meso Scale Diagnostic, cat. no. K151TGD) according to the manufacturer’s protocol. Both soluble and insoluble α-synuclein levels from cortex and hippocampus samples were evaluated in comparison to an adequate protein standard as pg human α-synuclein per μg total protein.

*MALDI HiPLEX IHC method*. Matrix-assisted laser desorption/ionization high-plex immunohistochemistry (MALDI HiPLEX IHC) was executed on cryosectioned frozen brain tissue samples according to previous protocols [34] using photocleavable mass-tags (PC-MTs) developed by Ambergen, Inc. (Billerica, MA). The PC-MT antibody neurology panel from Ambergen was used for tissue staining according to the manufacturer’s protocol. Tissue sections were then prepared for imaging mass spectrometry analysis. Matrix was applied to each tissue using the HTX TM Sprayer (HTX Technologies, Chapel Hill, NC) according to Ambergen MALDI HiPLEX IHC protocols. Following matrix application, a recrystallization step was performed by enclosing the tissue sections in a humidified chamber containing 5% IPA for 2 minutes at 55°C. Slides were then imaged using a Bruker timsTOF fleX MALDI-2 instrument (Bruker Daltonics, Bremen, Germany) at a spatial resolution of 20 μm. Data processing and image analysis was performed using SCiLS Lab (Bruker Daltonics, Bremen, Germany).

### Statistics

Unless otherwise stated in the text, statistical comparisons were generated using GraphPad Prism (Dotmatics, Boston, MA) using a one-way ANOVA with post hoc analysis using multiple comparisons vs vehicle.

## Results

To evaluate whether targeting ferroptosis through the inhibition of 15-LO might represent a compelling therapeutic approach by offering a long-term, neuroprotective intervention in PD, we conducted several physiologically relevant studies in vitro and in vivo using PTC-041. The effects of 15-LO inhibition on neurite protection and prevention of neuronal loss in vitro were assessed along with the ability of PTC-041 to mitigate α-synuclein pathology and rescue motor defects in preclinical models of PD.

### PTC-041 is a reductive lipoxygenase inhibitor

**Fig 2:**
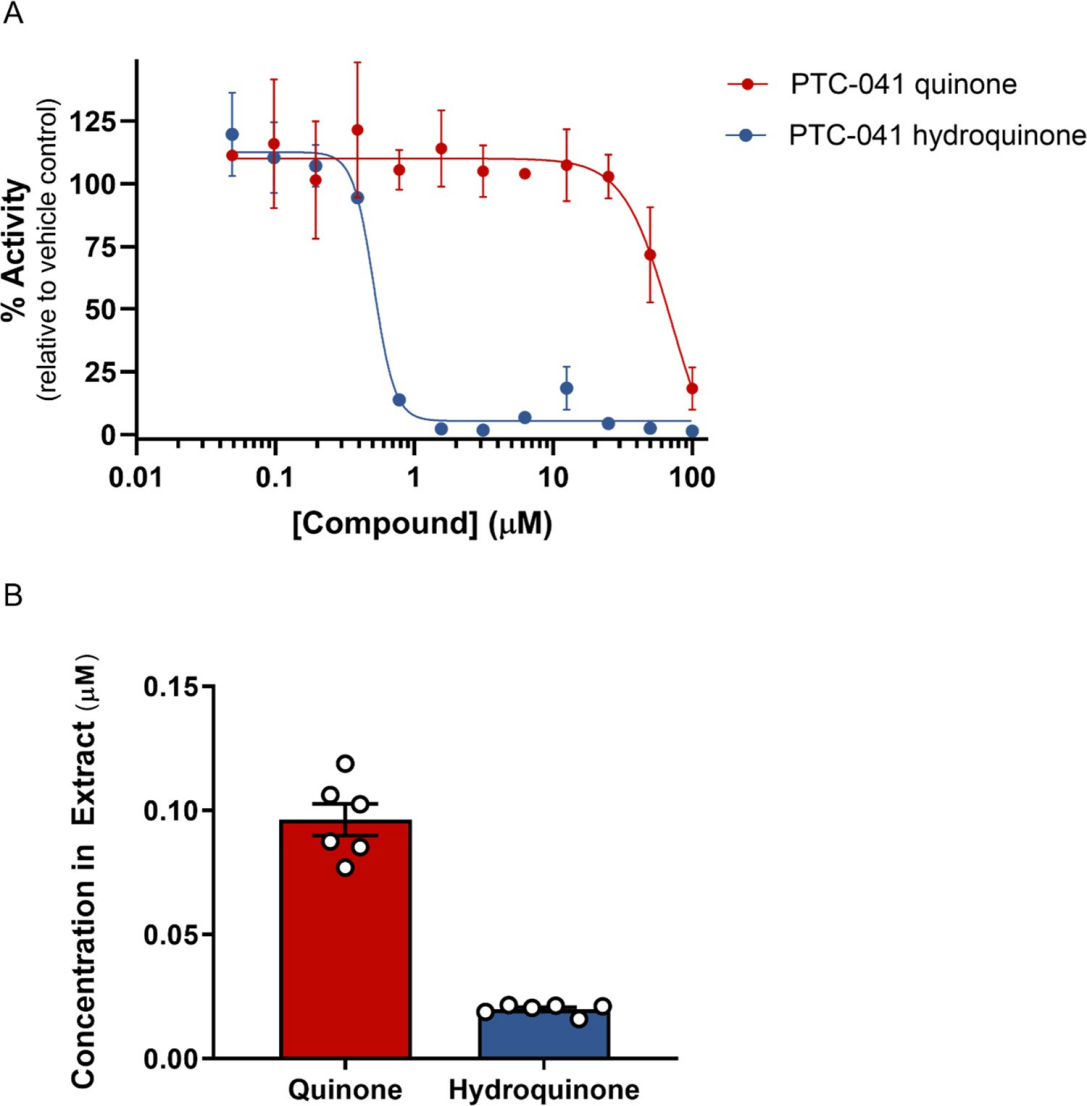
PTC-041 hydroquinone directly inhibits 15-lipoxygenase and is detected in primary rat neuron cultures. (**A**) Human 15-LO activity measured by liquid chromatography with tandem mass spectrometry (LC-MS) utilizing arachidonic acid (10 μM) as substrate. Mean ± SD (n = 4) values are displayed. Similar results were observed with rabbit 15-LO enzyme. Concentrations of PTC-041 quinone and hydroquinone measured by succinate capping method using mass spectroscopy 4 hours after PTC-041 quinone administration (1 μM) in DIV4 primary cortical neuron co-cultures. Data are presented as mean ± SEM of n = 6 wells/condition.

The LO inhibitory activity of PTC-041 was evaluated in its oxidized (quinone) and reduced (hydroquinone) forms (Fig 2A). Treatment of cells with PTC-041 quinone resulted in readily detectable steady-state levels of both the quinone and hydroquinone forms (Fig 2B). These data demonstrate the occurrence of the cellular conversion of the oxidized quinone to the reduced hydroquinone, suggesting that cellular reductive LO inhibition was plausible.

Table 2 shows that the reduced form is a more potent inhibitor of 15-LO activity than the oxidized form. PTC-041 hydroquinone preferentially inhibited human 15-LO and 5-LO compared to 12-LO and cyclooxygenase (COX-2).

**Table 2:** IC_50_ values–PTC-041 hydroquinone inhibition of human lipoxygenase and cyclooxygenase enzymes in vitro.

Enzyme	IC_50_ (μM)
COX-2	9.6^a^
5-LO	0.46^b^
12-LO	5.2^b^
15-LO	0.95^b^

IC_50_ was used as a potency metric; no direct assessment of K_d_ or K_i_ (via Cheng-Prusoff and a presumption of competitive inhibition) was made.

^a^ n = 4 (2 sets of independent duplicates)

^b^ n = 3.

### PTC-041 potently prevents ferroptotic cell death of PD patient-derived fibroblasts

To assess the anti-ferroptotic protective effects of PTC-041, we first established cellular assays using skin fibroblasts derived from 6 distinct patients with PD. Three of these cell lines were derived from patients with familial PD (fPD) harboring risk alleles for *LRRK2*, *PRKN*, or *MAPT*; the remaining lines were derived from patients with sporadic PD (sPD) (Table 3). To induce ferroptosis, each PD patient fibroblast culture was treated with the irreversible GPX4 inhibitor RSL3 [27], and cellular lipid oxidation, lipidomics, and viability were assessed over time.

**Table 3:** Potency of PTC-041 protection from ferroptosis in primary fibroblasts derived from donors with Parkinson’s disease.

Cell type	NINDS cell ID	Disease	Gene variant	Donor age at time of skin biopsy (y)	Age at diagnosis (y)	P_50_ (nM) Mean ± SD (n)
Primary skin fibroblasts	ND34263	fPD	*GBA* N370S	65	40	43 ± 6 (4)
	ND40070	fPD	*MAPT* N279K	43	43	62 ± 32 (15)
	ND40078	fPD	*PARK2* R275W/R275Q (compound)	51	47	80 ± 61 (5)
	ND30116	sPD	Unknown	67	47	60 ± 18 (5)
	ND33424	sPD	Unknown	57	47	30 ± 6 (4)
	ND33847	sPD	Unknown	53	51	29 ± 3 (4)
	All 6 donors combined [mean ± SD (n)]					51 ± 20 (6)

Cell survival was assessed by CTG 2.0 assay 24 hours after RSL3 (2 μM) and concomitant compound treatment (24-point dose response). Rescue potency was determined by standard 4-parameter curve fitting (Dotmatics), with the EC_50_ concentrations reported. The potency of each compound was determined in multiple independent experiments (range 4–15, median 4.5) in each of 6 different donors’ cells. **Abbreviations:** EC_50_, half-maximal effective concentration; fPD, familial Parkinson’s disease; NINDS, National Institute of Neurological Disorders and Stroke; SD, standard deviation; y, years; sPD, sporadic Parkinson’s disease.

**Fig 3:**
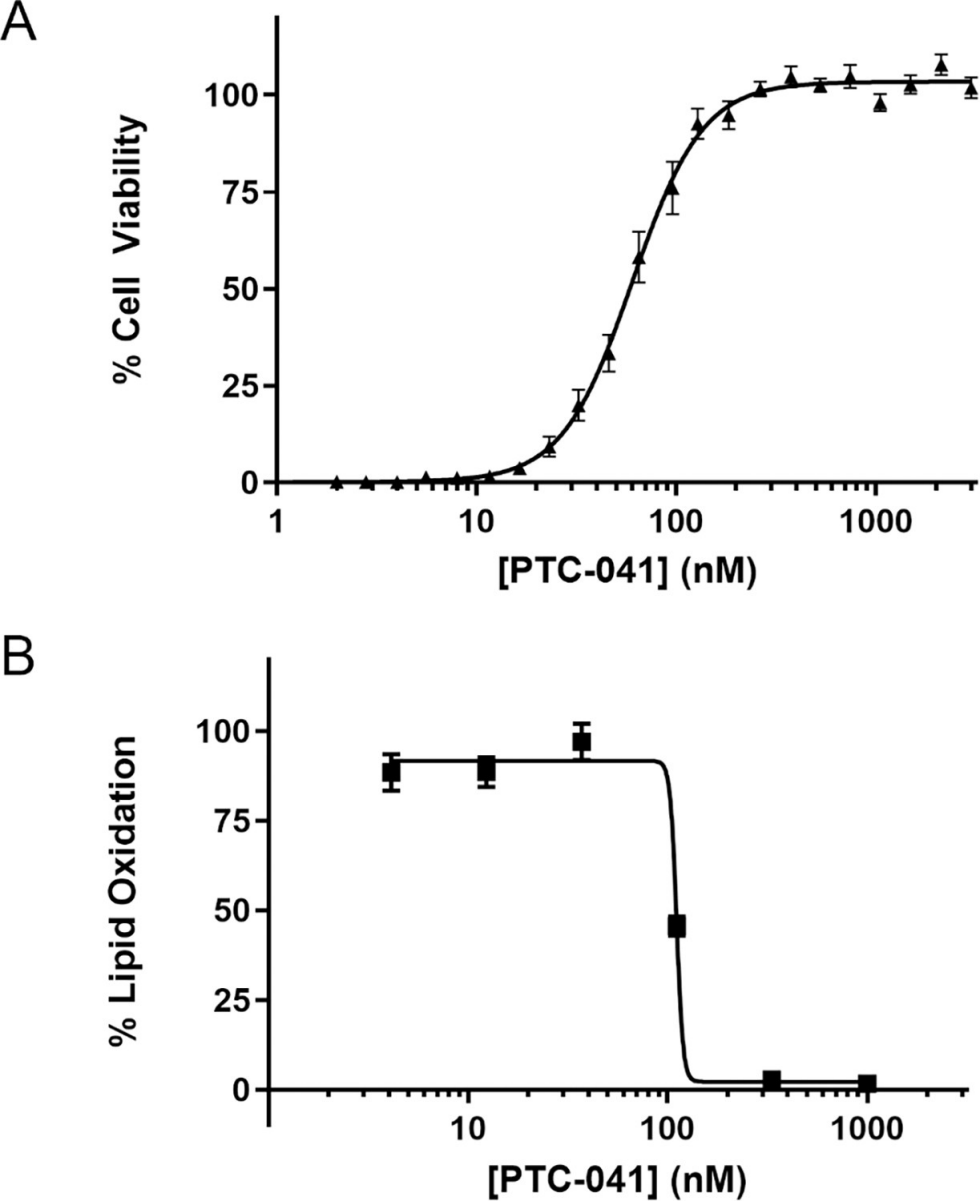
PTC-041 potently protects primary Parkinson’s disease patient-derived fibroblasts from ferroptotic lipid oxidation and cell death. (A) Cell survival was assessed by CTG 2.0 assay 24 hours after RSL3 (2 μM) and concomitant PTC-041 treatment (24-point dose response). Summary results for 1 representative donor’s cells (ND40070) are shown as mean ± SEM of multiple replicates (n = 15) from 4 independent experiments. (B) Cell lipid oxidation was assessed by monitoring the rate of change in total green fluorescence area per well of BODIPY C11 581/591-prelabeled cells as measured over time in the IncuCyte S3 instrument. Five hours after RSL3 (2 μM) and concomitant compound treatment (6-point dose response in experimental triplicate), the rescue potency was evaluated.

RSL3 treatment of PD patient fibroblasts induced a time-dependent increase in cell death and lipid oxidation as assessed by green fluorescence shift of BODIPY^TM^ C11 redox-sensitive lipid dye using time-lapse fluorescence video microscopy. Strikingly, PTC-041 treatment completely prevented ferroptotic cell death induced by RSL3, with a mean EC_50_ potency value of 80 nM (Fig 3A). PTC-041 also dose-dependently prevented RSL3-dependent lipid oxidation, with a mean potency (IC_50_) of 109 nM (Fig 3B). In addition, RSL3 treatment resulted in a >95% reduction in PD patient fibroblast viability at a maximally efficacious concentration. The mean PTC-041 EC_50_ potency value of all PD patient-derived fibroblasts tested was 51 nM, demonstrating near-maximal rescue in all cells tested at concentrations of ≥300 nM and suggesting efficacy across patient fibroblasts from different genetic backgrounds.

**Fig 4:**
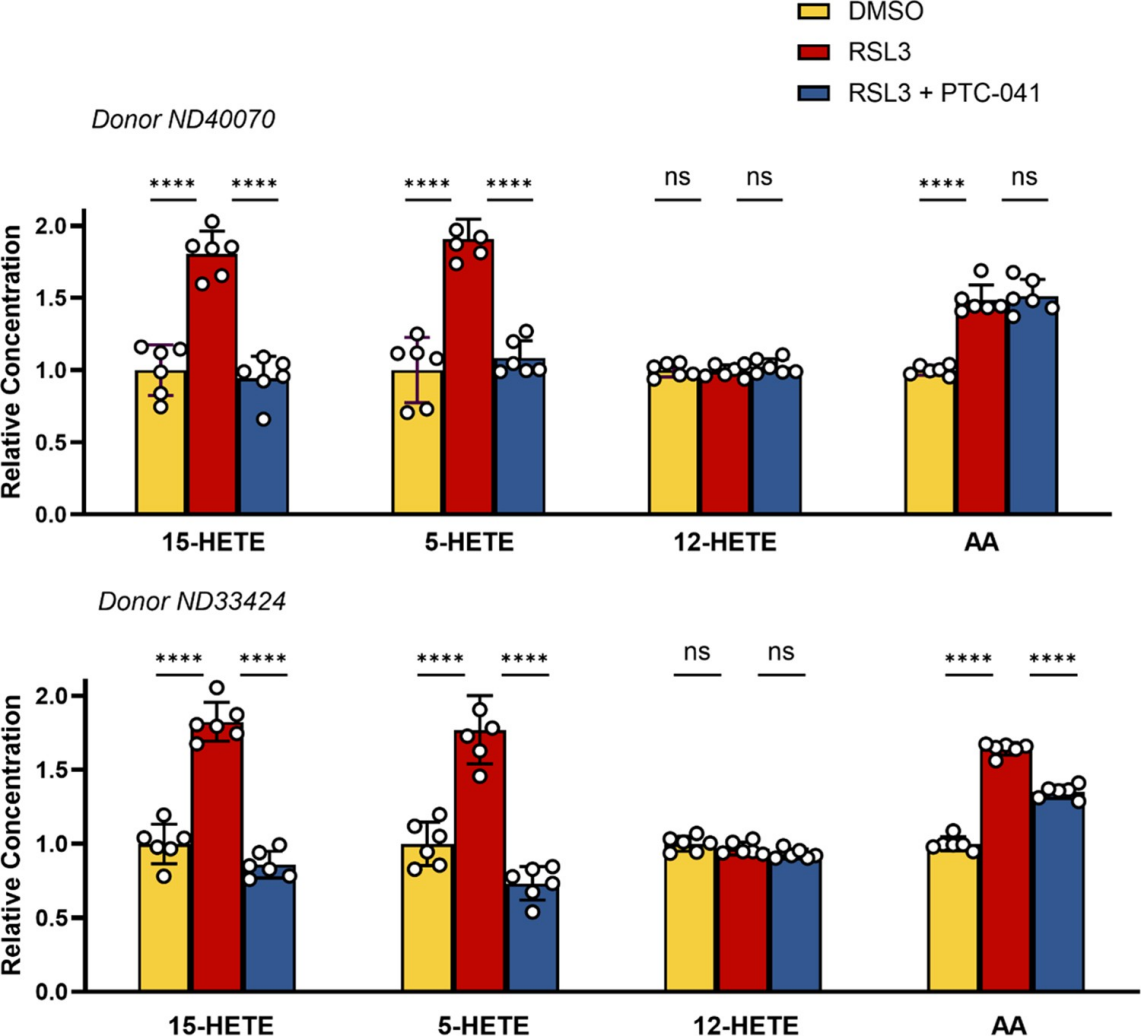
PTC-041 prevents 15-HETE release from RSL3-treated PD patient-derived fibroblasts. Patient-derived fibroblasts [(A) donor ND40070; (B) donor ND33424] were treated with RSL3 (2 μM) with or without concomitant PTC-041 treatment (ND40070, 700 nM; ND33424, 300 nM). After 5.5 to 6 hours of treatment, the cellular conditioned medium was collected and 15-, 5-, or 12-HETE or AA was quantitated using targeted LC-MS methods. Note that the cognate hydroperoxidated (HpETE) species were also analyzed but were below the limits of quantitation for >90% of the samples, preventing further analysis. Results shown are from 1 experiment per cell type, in which 6 independently treated wells were analyzed by LC-MS separately, with each LC-MS sample being analyzed in singlicate. Mean ± SD (n = 6) values are displayed. Statistical test applied: 1-way ANOVA compared to the RSL3-only group, with Dunnett’s test for multiple comparisons, where **** = p<0.0001, *** = p≤0.001, ** = p≤0.01, * = p≤0.05, and ns = p>0.05.

Lipidomic analysis of the conditioned medium from RSL3-treated PD patient-derived fibroblasts revealed a pronounced increase in both 5-HETE and 15-HETE, with no change in 12-HETE. PTC-041 co-treatment completely prevented RSL3-induced cellular 5/15-HETE release (Fig 4), which is consistent with the enzyme inhibition data (Table 3). In assessing the relative potency of PTC-041 and related compounds, PTC-041 was shown to be a potent anti-ferroptotic agent, with a mean RSL3 survival EC_50_ of 67 nM (S1 Table).

### PTC-041 inhibits ferroptotic but not other forms of regulated cell death in Q7 cells

**Fig 5:**
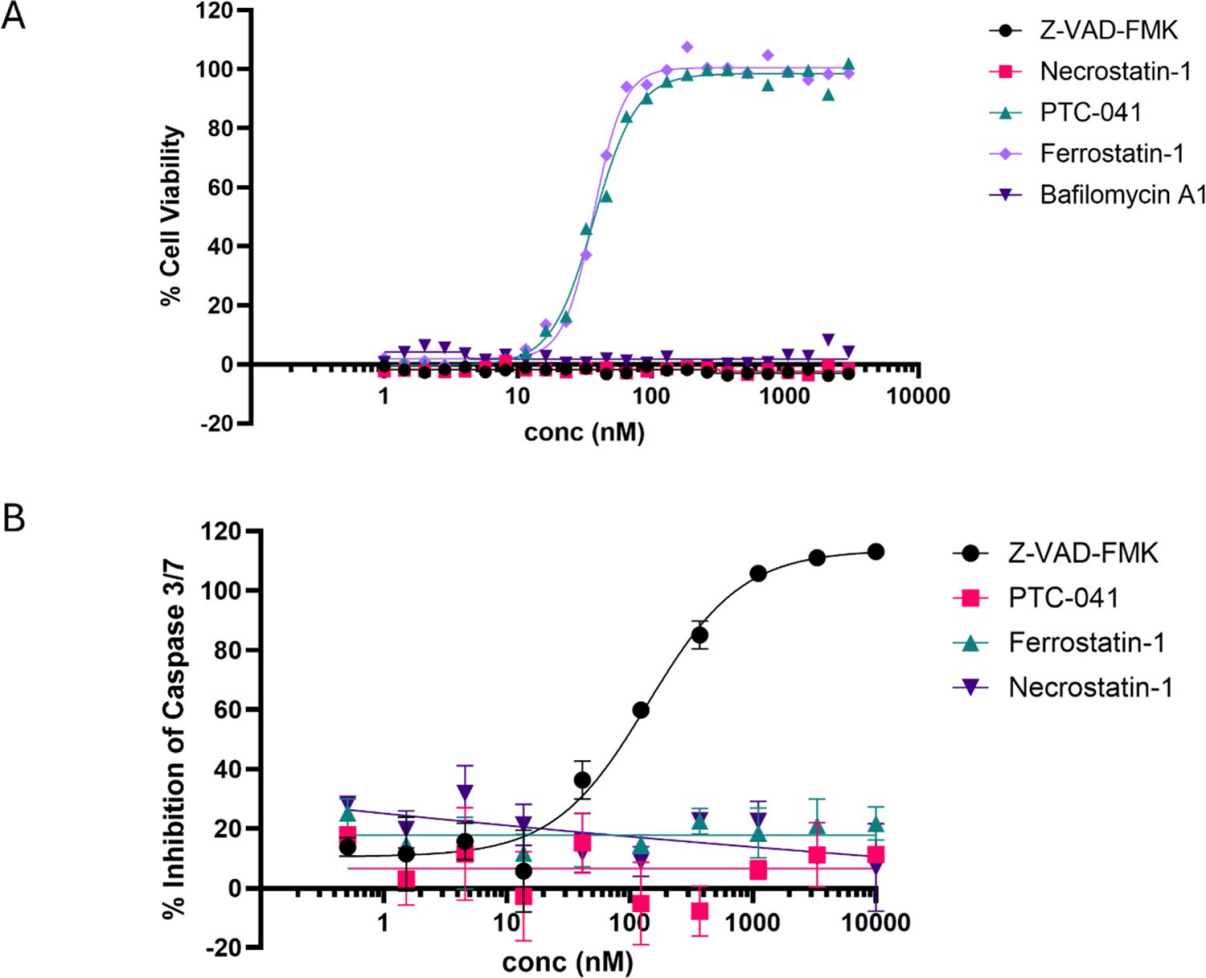
PTC-041 prevents ferroptosis but not other forms of regulated cell death. (A) Q7 cells were incubated for 15 minutes with PTC-041, ferrostatin-1 (inhibitor of ferroptosis), Z-VAD-FMK (inhibitor of apoptosis), necrostatin-1 (inhibitor of necroptosis), and bafilomycin A1 (autophagy inhibitor) at the concentrations indicated. Ferroptosis was induced with RSL3 (2 μM). Cells were incubated for 18 hours, and cell viability was assessed. Representative trace of 3 independent experiments. The EC_50_ values for cell viability were 37.21, 51.52, and 30.88 nM (PTC-041) and 37.01, 44.54, and 28.61 nM (ferrostatin-1). (B) Q7 cells were incubated for 15 minutes with PTC-041, ferrostatin-1 (inhibitor of ferroptosis), Z-VAD-FMK (inhibitor of apoptosis), and necrostatin-1 (inhibitor of necroptosis) at various concentrations. Caspase activation was induced with staurosporine (1 μM). Cells were incubated for 6 hours and caspase activity was assessed. Traces show representative of 5 independent experiments conducted.

The anti-ferroptotic properties of PTC-041 were compared with those of ferrostatin-1 (inhibitor of ferroptosis), Z-VAD-FMK (inhibitor of apoptosis), necrostatin-1 (inhibitor of necroptosis), and bafilomycin A1 (autophagy inhibitor). After a 15-minute pretreatment with inhibitors, ferroptosis was induced in Q7 cells with the irreversible GPX4 inhibitor, RSL3. Fig 5A shows that ferrostatin-1 and PTC-041 inhibited ferroptosis with similar potency, while classical inhibitors of apoptosis or necroptosis failed to prevent RSL3-dependent cell death. Conversely, ferroptotic compounds, including PTC-041 failed to prevent caspase activity, a canonical feature or apoptosis. For these studies, apoptosis was induced with staurosporine, and caspase activation was monitored using a caspase activity assay. The well-known apoptotic inhibitor Z-VAD-FMK was the only compound capable of inhibiting apoptosis (Fig 5B). Altogether these data demonstrate that PTC-041 selectively inhibits ferroptotic-mediated cell death.

### PTC-041 prevents primary neuronal neurite integrity loss and associated astrogliosis

We next assessed the activity and potency of PTC-041 in primary neuronal cultures from embryonic rat midbrain. The expression of lipoxygenase (*Alox*) genes was determined by quantitative reverse transcription polymerase chain reaction (qRT-PCR). Intriguingly, the most abundant LO gene expressed in primary rat midbrain neuronal cultures was determined to be arachidonate 15-lipoxygenase (*Alox15*), which encodes 15-LO, suggesting a role for the enzyme in neuronal physiology and/or pathology (S1 Fig).

**Fig 6:**
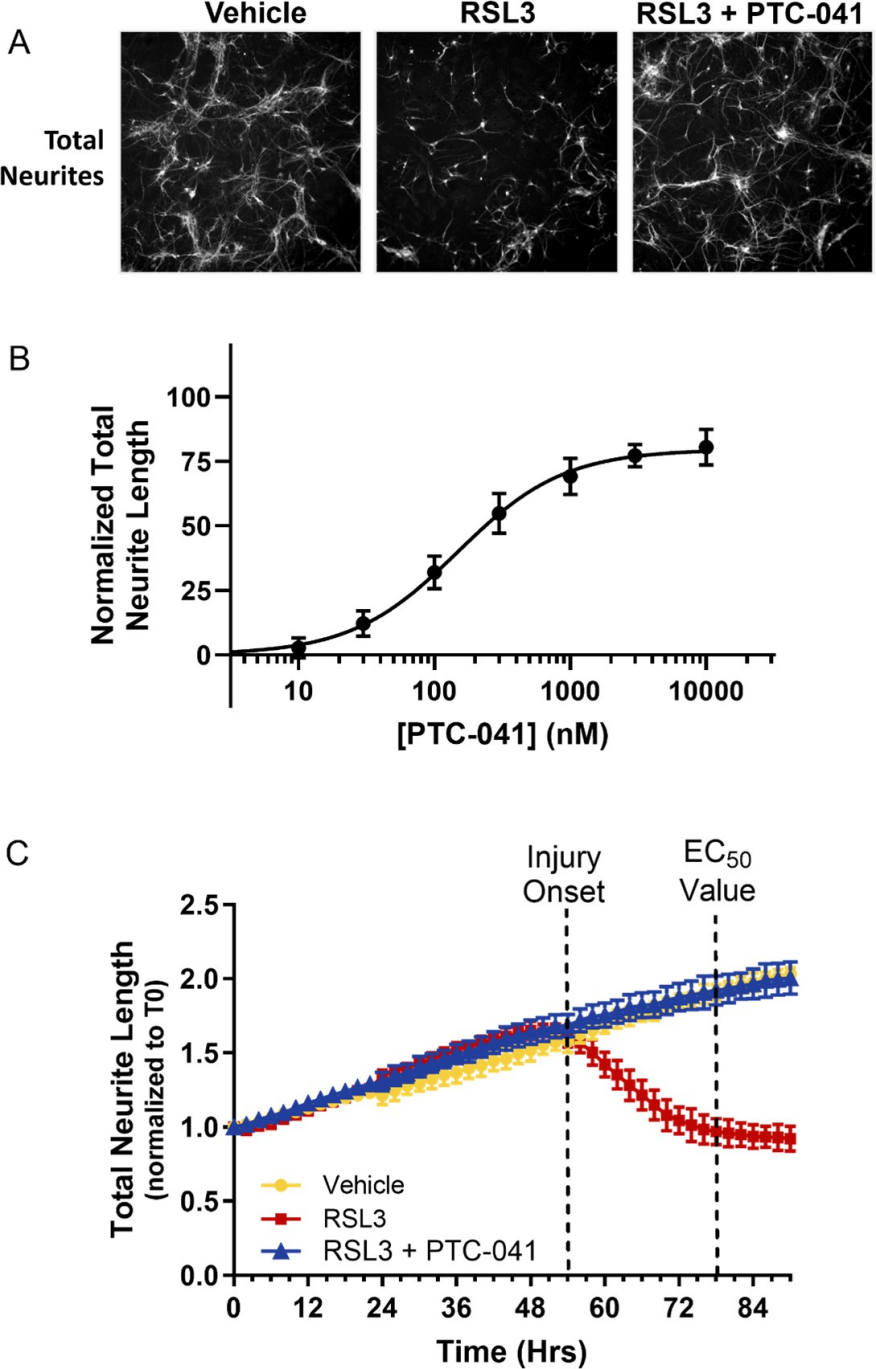
PTC-041 prevents neurite loss in primary rat midbrain neurons and prolongs neurite protection in response to RSL3 challenge. (A) Total neurite (MAP2) integrity was quantified with the ArrayScan XTI high-content imaging platform (ThermoFisher). Representative images for 1000 nM PTC-041 are shown. (B) Ferroptotic cell death was induced in primary rat midbrain neuronal cultures 24 hours after plating (DIV1) with the GPX4 inhibitor RSL3 (1.25 μM). Total neurite length was quantified over time with the IncuCyte platform. (C) Prolonged total neurite protection was observed on treatment with PTC-041 (300 nM). Neurite protection EC_50_ values were estimated at 24 hours after the onset of RSL3-induced neurite injury. All data are presented as mean ± SEM of n = 6 wells/condition.

PTC-041 co-treatment dose-dependently and completely prevented RSL3-induced neurite loss, with a mean EC_50_ of 148 nM (95% confidence interval 91 to 276 nM, combining data from 3 independent experiments; Fig 6A and 6B). Further, treatment of DIV1 primary rat midbrain neuronal cultures with RSL3 resulted in a time-dependent decline in neurite integrity, as assessed by time-lapse phase contrast microscopy (Fig 6C). PTC-041 demonstrated >90% rescue at concentrations of ≥1000 nM, suggesting a minimally efficacious concentration in neuronal cell systems.

### PTC-041 prevents ferroptosis-associated α-Synuclein accumulation, nitrosylation, and aggregation in vitro

**Fig 7:**
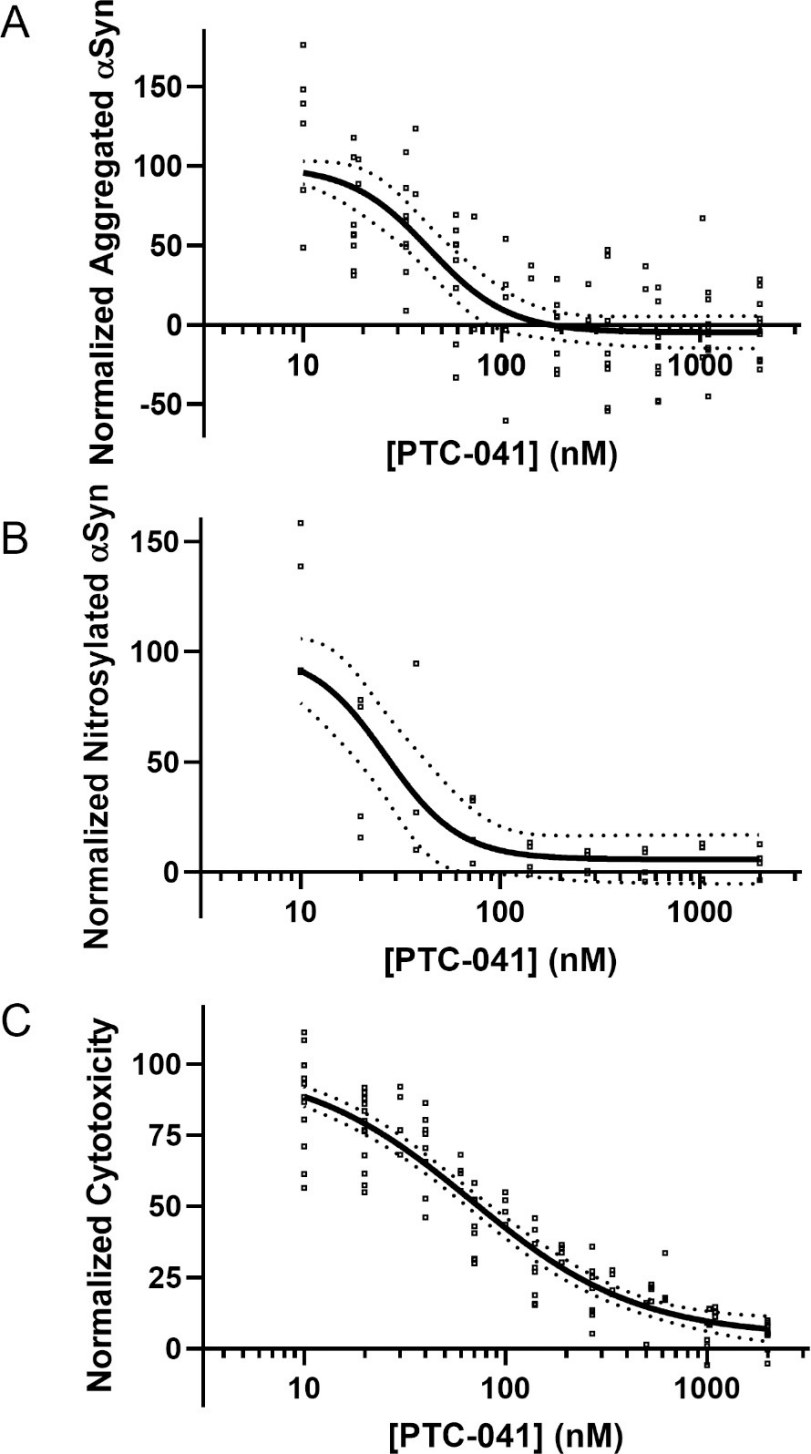
PTC-041 inhibits RSL3-induced α-synuclein aggregation and nitrosylation. (**A**) Aggregation: Results are the average of 3 independent experiments. Data were expressed relative to the average of the RSL3-only control wells (100%) and dimethylsulfoxide (DMSO)-only control wells (0%). Each dot corresponds to 1 well. The top of the curve was constrained to 100. Solid line indicates best-fit curve; dotted lines represent 95% confidence intervals for best-fit curve. Best-fit values (95% confidence intervals): IC_50_, 43 nM (32 to 58 nM); Bottom, -5% (-15% to 5%); Hill slope, -2.2 (-3.9 to -1.3). (**B**) Nitrosylation: Results are the average of 2 independent experiments. Data were expressed relative to the average of the RSL3-only control wells (100%) and DMSO-only control wells (0%). Each dot corresponds to 1 well. The top of the curve was constrained to 100. Solid line indicates best-fit curve; dotted lines represent 95% confidence intervals for best-fit curve. Best-fit values (95% confidence intervals): IC_50_, 27 nM (16 to 43 nM); Bottom, 6% (-7% to 19%); Hill slope, not calculable. (**C**) Cytotoxicity: Results are the average from 3 independent experiments. Data were expressed relative to the average of the RSL3-only control wells (100%) and DMSO-only control wells (0%). Each dot corresponds to 1 well. Curve top was constrained to 100. Solid line indicates best-fit curve; dotted lines represent 95% confidence intervals for best-fit curve. Best-fit values (95% confidence intervals): IC_50_, 68 nM (55 to 87 nM); Bottom, 4% (-3% to 10%); Hill slope, -1.0 (-1.3 to -0.9).

Several lines of genetic and biochemical evidence accumulated over the past 30 years implicate lipid oxidation and oxidative stress in the pathology of PD. More recently, potential reciprocal feed-forward links between iron accumulation, lipid oxidation, and α-synuclein post-translational changes leading to aggregation have been proposed [21, 35–38]. Given this potential interplay, we generated N27 cells ectopically expressing a human α-synuclein-GFP fusion protein and assessed the effects of RSL3-mediated GPX4 inhibition on α-synuclein. After RSL3 treatment, we observed α-synuclein aggregation and α-synuclein nitrosylation, followed by cell death. These results are consistent with the hypothesis that increased levels of lipid oxidation associated with ferroptosis have pathogenic effects on the state of α-synuclein aggregation. Critically, we observed that PTC-041 was able to prevent all RSL3-induced changes in α-synuclein aggregation (Fig 7A), nitrosylation (Fig 7B), and cell death (Fig 7C), with IC_50_ potencies ranging from 17 to 77 nM. These observations are consistent with recent research indicating that the ferroptosis pathway sits at the nexus of oxidative stress and (neuro)inflammation [28].

### PTC-041 lowers pathological α-synuclein and protects cellular viability in vivo

**Fig 8:**
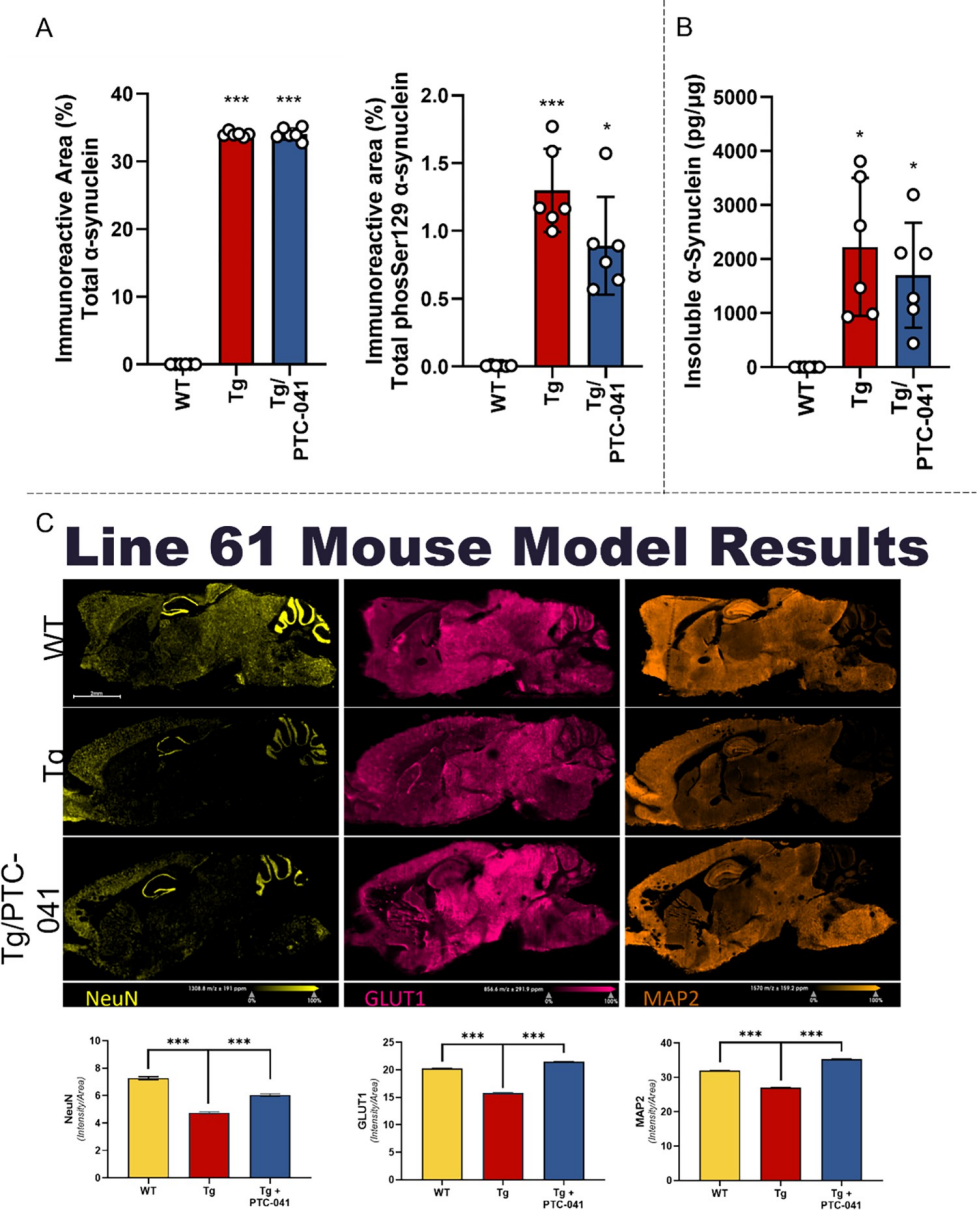
Treatment of Line 61 transgenic mice with PTC-041 results in attenuated α-synuclein pathology. (**A**) Quantification of total and pSer129 α-synuclein immunofluorescence in the cerebral isocortex. Graphs present the means of immunofluorescent signal measured within the region of interest on 5 brain sections per mouse (n = 6 per group). Bar graphs represent group means ± SEM (n = 6 per group); ***p<0.0001. Data were analyzed by one-way analysis of variance (ANOVA) and Dunnett’s post hoc test for multiple comparisons; WT (Vehicle) group was defined as statistical control for comparisons. **(B**) Quantification of insoluble human α-synuclein levels in the cortex. Data are displayed as pg α-synuclein per μg total protein; bar graphs represent group means ± SEM (n = 6 per group); ***p<0.0001, **p<0.001, *p<0.05. Data were analyzed by one-way ANOVA and Dunnett’s post hoc test for multiple comparisons; WT (Vehicle) group was defined as statistical control for comparisons. **(C**) MALDI-IHC was used to image proteins in sagittal brain sections of wild-type (WT), Line 61 transgenic (Tg), and PTC-041-dosed transgenic (Tg + PTC-041) mice. Loss of neurons (NeuN ●), non-neuronal cells expressing Glut1 (●), and neurites expressing MAP2 (●) were also observed in Line 61 transgenic mice relative to WT, and PTC-041 was able to rescue this loss. Bar graphs represent means of MALDI-IHC signal measured ± SEM, n = 3; ***p<0.0001.

To validate the observation that PTC-041 can mitigate pathological α-synuclein in vitro, we administered PTC-041 to 6-week-old transgenic Line 61 mice daily for 6 weeks and then evaluated the effect of the compound on pathological changes in α-synuclein. This well-characterized mouse model overexpresses human α-synuclein under regulation by the Thy-1 promoter and reproduces several aspects of sporadic PD [39, 40]. Animals display proteinase-K -resistant α-synuclein aggregates as early as 1 month and neuronal loss around 3 months, making it a convenient model to evaluate compound effects on α-synuclein pathology. After 6 weeks of dosing, we found that PTC-041 did not alter the robust increase in total α-synuclein observed, but it induced a marked trend towards reduction in aggregated and phosphorylated α-synuclein (Fig 8A and 8B).

•To evaluate the effect of PTC-041 on neuronal viability in older mice, a separate study was conducted wherein 6-week-old transgenic Line 61 mice were dosed with PTC-041 daily for 15 weeks. At the end of this 15-week period, matrix-assisted laser desorption/ionization immunohistochemistry (MALDI-IHC) was used to image proteins in sagittal brain sections of wild-type (WT), Line 61 transgenic (Tg), and PTC-041-dosed transgenic (Tg + PTC-041) mice (Fig 8C). Decreases in NeuN and Map2 in Line 61 brains suggest a loss of neurons and dendrites, particularly in the hippocampus and cerebellum. This loss was either partially or fully restored with PTC-041 treatment. Loss of Glut1, a glucose transporter widely distributed throughout the brain and predominantly observed in endothelial cells and astrocytes, suggests that the loss of neurons also affects non-neuronal cells. Again, treatment with PTC-041 restores Glut1 expression in the transgenic mice.

### PTC-041 protects against 6-OHDA-induced motor deficits in hemiparkinsonian rat model

To further evaluate the protective effects of PTC-041 against PD-associated functional impairments in vivo, we examined the efficacy of PTC-041 in a 6-hydroxydopamine (6-OHDA) rat model of PD. This hemiparkinsonian rat model was generated by unilateral injection of 6-OHDA neurotoxin into the MFB. Stereotaxic delivery of 6-OHDA into the MFB results in ROS production and subsequent degeneration of dopaminergic neurons in the ventral tegmental area and substantia nigra pars compacta (SNpc) [41]. This lesion recapitulates the major aspects of human PD pathology, including the degeneration of dopaminergic neuronal pathways and motor impairments, and is characterized using rotational analysis with apomorphine challenge and cylinder test [32]. Approximately 2 weeks after unilateral injection of vehicle or 6-OHDA followed by daily treatment with sesame oil or PTC-041, animals were subjected to the cylinder test and apomorphine-induced circling (rotation) test to assess motor function.

#### Cylinder test

**Fig 9:**
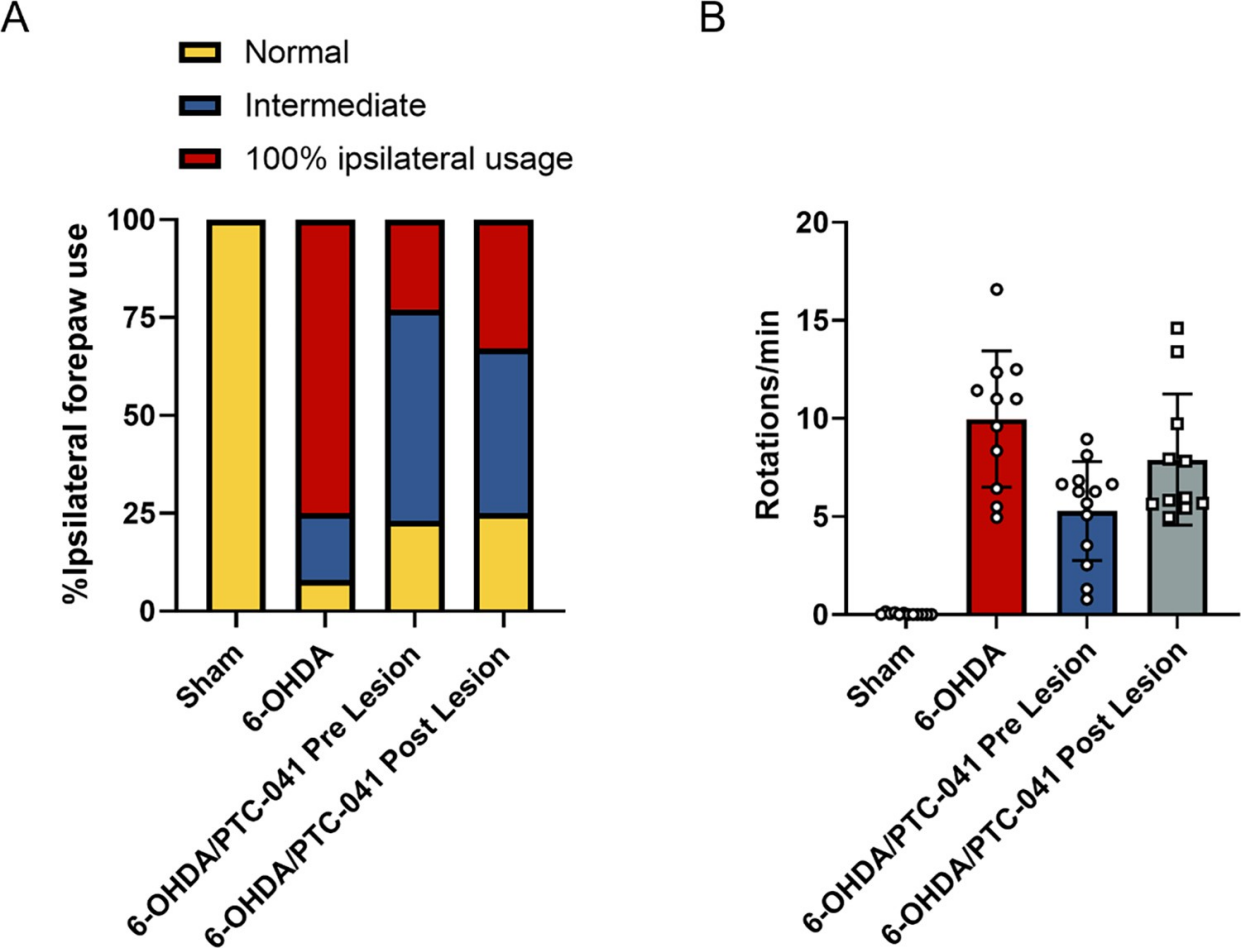
PTC-041 improves motor deficits caused by dopaminergic neuron loss. 6-OHDA lesion recapitulates the major aspects of the PD phenotype as measured by cylinder and rotation tests. (**A**) Cylinder test. 6-OHDA-lesioned animals demonstrate profound forelimb use asymmetry, with >80% preference for the ipsilateral paw indicating >95% dopaminergic neuron loss. Normal (yellow) represents the percentage of animals where scoring of forelimb usage was within 2 SD of the mean of the Sham group. Intermediate (blue) represents the percentage of animals that displayed preferential ipsilateral forepaw use between 2 SD and 3 SD above the mean of the Sham group. 100% ipsilateral usage (red) represents the percentage of animals that relied solely on their ipsilateral paw. (**B**) Apomorphine-induced rotation test. Based on the literature, an increase in contralateral rotations of more than 5 per minute is the minimum threshold indicating PD pathology [32]. Bars show the mean (± SEM) number of contralateral turns per minute for each group. Individual markers represent individual animal values.

We found the majority of sesame oil-dosed 6-OHDA-lesioned rats demonstrated 100% ipsilateral paw preference, indicating >95% dopaminergic neuron loss in the SNpc. PTC-041 treatment at 24 hours both pre- and post-lesion resulted in a normalization of the forelimb asymmetry (Fig 9A).

#### Rotational analysis with apomorphine challenge

Overall, 6-OHDA-lesioned rats that received sesame oil showed rotation scores (in contralateral turns per minute) that were, on average, higher than the vehicle control group, demonstrating significant functional deficits due to 6-OHDA injection. Treatment with PTC-041 resulted in decreased the number of contralateral rotations per minute of 6-OHDA-lesioned animals after apomorphine challenge, with significant reductions in contralateral turns observed for 6-OHDA-lesioned animals that received PTC-041 24 hours pre-lesion (Fig 9B).

## Discussion

The progressive nature of PD and the debilitating features of advanced stages of the disease highlight an unmet medical need to develop new medicines that arrest or modify the underlying mechanisms of neuronal cell death. Recent research has identified ferroptosis to be a driver in PD pathogenesis [10, 11, 42], and emerging evidence supports the role of downstream LO products (HETEs) as central in ferroptotic cell death [28, 43]. Thus, targeting these LO enzymes to prevent ferroptotic cell death offers entry into a new class of disease-modifying PD therapies. While lipoxygenase inhibition has previously been investigated as a therapeutic strategy in the neurological disease space, the body of work investigating 15-LO and its role in neurodegenerative disease remains relatively small [44–47]. Moreover, there are limited examples of therapies targeting 15-LO as a means of suppressing or preventing ferroptosis for the treatment of neurological diseases [28, 29]. To our knowledge, this work represents the first example of the development and characterization of an anti-ferroptotic 15-LO inhibitor for the treatment for PD.

Building on our understanding of the mechanism of action of the tocotrienol (vitamin E) paraquinone metabolite vatiquinone (also referred to as PTC743 or EPI-743) [28, 29], we sought to identify and develop a fully synthetic compound with similar activity and increased potency, which resulted in the selection of PTC-041 for further development.

We show that the protective effects of PTC-041 are mediated by selective inhibition of the ferroptotic pathway. Ferroptosis is a cell death process that, unlike apoptosis or necrosis, can also be initiated by the failure of reduced glutathione (GSH)-mediated antioxidant cellular defense [48]. GSH is an essential intermediary metabolite and important cofactor for the enzyme GPX4, which regulates ferroptosis by catalyzing the reduction of lipid hydroperoxides to lipid alcohols. After demonstrating that PTC-041 hydroquinone is a potent and selective LO inhibitor with preferential activity against human 15- and 5-LO, we validated its activity in cell models of PD where ferroptosis can be induced via irreversible chemical inhibition of GXP4 using small molecules such as RSL3 [49, 50]. Patient-derived fibroblasts with known PD risk alleles (*LRRK2*, *PRKN*, *or MAPT)* or fibroblasts derived from patients with sporadic PD [51] were treated with RSL3 to induce time-dependent accumulation of oxidized lipids, a consequence of increased HETE production and LO activation. Quantitative lipidomic analysis revealed both 5- and 15-HETE were robustly elevated, strongly supporting RSL3-dependent LO activation. Significant losses in cellular viability, with >95% of fibroblasts observed dead within 24 hours of RSL3 exposure, were also noted. Consistent with the lack of effect on 12-LO, levels of 12-HETE were not altered. Co-addition of PTC-041 dose-dependently prevented increased lipid oxidation and cell death induced by GPX4 inhibition.

To evaluate PTC-041 protection in a more disease-relevant cell system, we generated primary neuronal cultures from rat midbrain. We determined that this culture system expresses LO genes with the most abundant LO gene expressed being *Alox15* (15-LO; S1 Fig). Treatment of these primary neuronal co-cultures with RSL3 at DIV1 induced neurite loss, astrogliosis, and neuronal cell death, indicating that this cell system is susceptible to ferroptotic-mediated cell death. In the presence of PTC-041, we saw potent rescue of neurite integrity and prevention of neuronal cell death and astrogliosis following RSL3 treatment. These data strongly support the hypothesis that PTC-041 potently prevents ferroptotic-mediated cell death in primary neuronal co-cultures.

Lewy bodies (LBs), a key pathological hallmark of PD [52, 53], result from abnormal protein aggregates of α-synuclein. Single nucleotide polymorphisms and copy number variations in the *SNCA* gene, which encodes the α-synuclein protein, lead to increased protein aggregation and LB formation and are strongly associated with increased risk for idiopathic PD [54, 55]. Furthermore, there is emerging evidence that accumulation of oxidized lipids coincident with ferroptotic signaling contributes to α-synuclein aggregation [12, 21, 56]. It has been shown that α-synuclein oligomers are able to insert into membranes of human neurons, resulting in altered membrane conductance, disruption of normal calcium ion influx, and ultimately neuronal cell death. The peroxidation of PUFAs is hypothesized to modulate these membrane-aggregate interactions, specifically by increasing membrane susceptibility to α-synuclein insertion and ion flux alteration [12].

To evaluate whether PTC-041-derived inhibition of lipid oxidation might mitigate α-synuclein aggregation, we generated N27 cells that stably express human α-synuclein. In these cells overexpressing α-synuclein, addition of RSL3, as anticipated, caused a significant increase in aggregated α-synuclein and loss of cell viability. This increase in aggregation also corresponded with significant increases in detectable phosphorylated and nitrosylated α-synuclein which are well-established post-translational modifications associated with synuclein pathology [57, 58]. Strikingly, we observed that co-treatment with PTC-041 dose-dependently prevented the increases in aggregated α-synuclein and prevented increases in nitrosylated α-synuclein. Taken together, these data strongly support the recent observations that oxidized lipid accumulation downstream of ferroptosis contributes to synuclein pathologies associated with PD [12]. When we examined the potential neuroprotective effects of PTC-041 against α-synuclein pathology in the Thy1‑aSyn (Line 61) mouse model, we found a similar trend towards lowering of aggregated and phosphorylated α-synuclein, thereby validating our in vitro data. We further noticed that treatment with PTC-041 either partially or fully restored neuronal and dendritic losses observed in untreated mice, which suggests the neuroprotective capacity of anti-ferroptotic LO inhibitors in α-synuclein pathology.

Finally, we evaluated the efficacy of PTC-041 in vivo using the well-established 6-OHDA hemiparkinsonian rat model. It has been demonstrated that 6-OHDA-lesioned animals show marked forelimb use asymmetry when compared to the sham control group, with over 80% preference for the ipsilateral forepaw observed [32, 33, 41]. In line with these data, we found the majority of 6-OHDA-lesioned animals demonstrated 100% preference for the ipsilateral forepaw, indicating >95% unilateral dopaminergic neuron loss. Daily treatment with PTC-041 starting 24 hours post-lesion until the time of functional testing (2 weeks post-lesion) revealed clear improvements in forelimb use asymmetry, with most treated animals displaying reduced preference for the ipsilateral paw (between 75% and 95% preference). Furthermore, daily treatment starting 24 hours pre-lesion demonstrated similar functional improvements, which highlights the potential neuroprotective properties of PTC-041. Mechanistically, a loss of dopaminergic neurons has been widely described as a major consequence of injection of 6-OHDA in the MFB in rat models of PD [59–62]. The resulting dopamine depletion is responsible for the behavioral changes [63]. In this context, the symptomatic improvements observed under PTC-041 treatment are likely to be the result of preservation of dopaminergic neurons in the lesioned area.

The strong evidence linking ferroptosis to PD pathology has coincided with the development of new ferroptosis-targeting therapies for treating PD, including Ferriprox (deferiprone) [64] and ebselen [65, 66]. Additionally, the development of another small molecule inhibitor of ferroptosis, dl-3-n-butylphthalide, was recently reported which prevented ferroptotic cell death in an in vitro model of PD [26]. Of these, only deferiprone, which functions as an iron chelator to sequester Fe(III) and works downstream from the pro-ferroptotic hydroperoxide products of 15-LO, has advanced into clinical trials. Similarly, none of the other proposed anti-ferroptotic compounds directly target ferroptotic LOs, rendering PTC-041, as a reductive LO inhibitor, unique among these therapies. Furthermore, deferiprone and these other compounds depend on mechanisms of action that are stoichiometrically limited, which compromises their potency at lower doses. In contrast, we compared PTC-041 to both deferiprone and ebselen and found PTC-041 showed an approximately 450-fold increase in anti-ferroptotic potency, indicating the potential for PTC-041 to be therapeutically effective at much lower doses (S1 Table).

In conclusion, we described here the development and characterization of PTC-041, a potent anti-ferroptotic therapeutic for the treatment of PD. Given the reported role of oxidative stress and ferroptosis in PD pathology and the promising therapeutic properties of PTC-041, the continued development of PTC-041 and other LO inhibitors for the treatment of PD and related neurodegenerative disorders with a common ferroptotic pathology is warranted.

## Supporting information

S1 Fig Lipoxygenase isoform mRNA expression profile in primary rat midbrain and cortical neuron co-culture systems. Alox gene expression levels were detected by qRT-PCR in primary rat midbrain neuron co-cultures at DIV1. All data are presented as mean ± SEM of n = 3 wells/condition, n = 1 experiment.
(TIF)

S1 Table Anti-ferroptotic potency of PTC-041 compared to other compounds. Cell survival was assessed by CTG 2.0 assay 24 hours after RSL3 (2 μM) and concomitant compound treatment (24-point dose response). Rescue potency was determined by standard 4-parameter curve fitting (Dotmatics), with the concentration for half-maximal rescue activity (EC50) reported here. The potency of each compound was determined in 3 independent patient fibroblast cultures (ND40070, ND40078, and ND30116). The mean and SEM across the 3 donors’ cells were calculated.
(DOCX)

S1 Data (XLSX)

## References

[1] Feigin VL, Nichols E, Alam T, Bannick MS, Beghi E, Blake N, et al. Global, regional, and national burden of neurological disorders, 1990–2016: a systematic analysis for the Global Burden of Disease Study 2016. The Lancet Neurology. 2019;18(5):459–80. doi: 10.1016/S1474-4422(18)30499-X 30879893 PMC6459001

[2] Van Den Eeden SK, Tanner CM, Bernstein AL, Fross RD, Leimpeter A, Bloch DA, Nelson LM. Incidence of Parkinson’s Disease: Variation by Age, Gender, and Race/Ethnicity. Am J Epidemiol. 2003;157(11):1015–22. doi: 10.1093/aje/kwg068 12777365

[3] Dorsey ER, Bloem BR. The Parkinson pandemic—a call to action. JAMA neurology. 2018;75(1):9–10. doi: 10.1001/jamaneurol.2017.3299 29131880

[4] Mari Z, Mestre TA. The disease modification conundrum in parkinson’s disease: failures and hopes. Frontiers in Aging Neuroscience. 2022;14. doi: 10.3389/fnagi.2022.810860 35296034 PMC8920063

[5] Devos D, Hirsch E, Wyse R. Seven solutions for neuroprotection in Parkinson’s disease. Movement Disorders. 2021;36(2):306–16. doi: 10.1002/mds.28379 33184908

[6] Wenzel SE, Tyurina YY, Zhao J, St Croix CM, Dar HH, Mao G, et al. PEBP1 Wardens Ferroptosis by Enabling Lipoxygenase Generation of Lipid Death Signals. Cell. 2017;171(3):628–41.e26. Epub 2017/10/21. doi: 10.1016/j.cell.2017.09.044 ; PubMed Central PMCID: PMC5683852. 29053969 PMC5683852

[7] Ye LF, Stockwell BR. Transforming Lipoxygenases: PE-Specific Enzymes in Disguise. Cell. 2017;171(3):501–2. doi: 10.1016/j.cell.2017.10.006 29053966 PMC5960801

[8] Stockwell BR, Friedmann Angeli JP, Bayir H, Bush AI, Conrad M, Dixon SJ, et al. Ferroptosis: A Regulated Cell Death Nexus Linking Metabolism, Redox Biology, and Disease. Cell. 2017;171(2):273–85. Epub 2017/10/07. doi: 10.1016/j.cell.2017.09.021 ; PubMed Central PMCID: PMC5685180. 28985560 PMC5685180

[9] Lewerenz J, Ates G, Methner A, Conrad M, Maher P. Oxytosis/Ferroptosis—(Re-) Emerging Roles for Oxidative Stress-Dependent Non-Apoptotic Cell Death in Diseases of the Central Nervous System. Front Neurosci. 2018;12:214. doi: 10.3389/fnins.2018.00214 29731704 PMC5920049

[10] Do Van B, Gouel F, Jonneaux A, Timmerman K, Gele P, Petrault M, et al. Ferroptosis, a Newly Characterized Form of Cell Death in Parkinson’s Disease That Is Regulated by PKC. Neurobiology of disease. 2016;94:169–78. Epub 2016/05/18. doi: 10.1016/j.nbd.2016.05.011 . 27189756

[11] Guiney SJ, Adlard PA, Bush AI, Finkelstein DI, Ayton S. Ferroptosis and cell death mechanisms in Parkinson’s disease. Neurochemistry international. 2017;104:34–48. Epub 2017/01/14. doi: 10.1016/j.neuint.2017.01.004 . 28082232

[12] Angelova PR, Choi ML, Berezhnov AV, Horrocks MH, Hughes CD, De S, et al. Alpha Synuclein Aggregation Drives Ferroptosis: An Interplay of Iron, Calcium and Lipid Peroxidation. Cell Death and Differentiation. 2020;27(10):2781–96. Epub 2020/04/29. doi: 10.1038/s41418-020-0542-z ; PubMed Central PMCID: PMC7492459. 32341450 PMC7492459

[13] Lan T, Sun TT, Wei C, Cheng T, Yang F, Zhang J-N, Li Q. Epigenetic Regulation of Ferroptosis in Central Nervous System Diseases. Molecular Neurobiology. 2023:1–16. doi: 10.1007/s12035-023-03267-1 36847936

[14] Liu L, Cui Y, Chang YZ, Yu P. Ferroptosis-related factors in the substantia nigra are associated with Parkinson’s disease. Scientific reports. 2023;13(1):15365. Epub 20230916. doi: 10.1038/s41598-023-42574-4 ; PubMed Central PMCID: PMC10505210. 37717088 PMC10505210

[15] Murphy RC, Bowers RC, Dickinson J, Zemski Berry K. Perspectives on the Biosynthesis and Metabolism of Eicosanoids. The Eicosanoids. 2004:1–16.

[16] Feng H, Stockwell BR. Unsolved Mysteries: How Does Lipid Peroxidation Cause Ferroptosis? PLoS biology. 2018;16(5):e2006203. Epub 2018/05/26. doi: 10.1371/journal.pbio.2006203 ; PubMed Central PMCID: PMC5991413. 29795546 PMC5991413

[17] Liddell J, White A. Nexus Between Mitochondrial Function, Iron, Copper and Glutathione in Parkinson’s Disease. Neurochem Int. 2018;117:126–38. doi: 10.1016/j.neuint.2017.05.016 28577988

[18] Moreau C, Duce JA, Rascol O, Devedjian J-C, Berg D, Dexter D, et al. Iron as a Therapeutic Target for Parkinson’s Disease. Movement disorders: official journal of the Movement Disorder Society. 2018;33(4):568–74. doi: 10.1002/mds.27275 29380903

[19] Sian J, Dexter D, Lees A, Daniel S, Agid Y, Javoy-Agid F, et al. Alterations in Glutathione Levels in Parkinson’s Disease and Other Neurodegenerative Disorders Affecting Basal Ganglia. Annals of Neurology. 1994;36:348–55. doi: 10.1002/ana.410360305 8080242

[20] Marttila RJ, Lorentz H, Rinne UK. Oxygen Toxicity Protecting Enzymes in Parkinson’s Disease. J Neurol Sci. 1998;86:321–31. 10.1016/0022-510x(88)90108-6 3221244

[21] Angelova PR, Horrocks MH, Klenerman D, Gandhi S, Abramov AY, Shchepinov MS. Lipid Peroxidation Is Essential for Alpha-Synuclein-Induced Cell Death. J Neurochem. 2015;133(4):582–9. Epub 2015/01/13. doi: 10.1111/jnc.13024 ; PubMed Central PMCID: PMC4471127. 25580849 PMC4471127

[22] de Farias CC, Maes M, Bonifacio KL, Bortolasci CC, de Souza Nogueira A, Brinholi FF, et al. Highly Specific Changes in Antioxidant Levels and Lipid Peroxidation in Parkinson’s Disease and Its Progression: Disease and Staging Biomarkers and New Drug Targets. Neuroscience Letters. 2016;617:66–71. Epub 2016/02/11. doi: 10.1016/j.neulet.2016.02.011 . 26861200

[23] Sanchez-Guajardo V, Tentillier N, Romero-Ramos M. The Relation Between α-Synuclein and Microglia in Parkinson’s Disease: Recent Developments. Neuroscience. 2015;302:47–58. 25684748 10.1016/j.neuroscience.2015.02.008

[24] Joers V, Tansey M, Mulas G, Carta A. Microglial Phenotypes in Parkinson’s Disease and Animal Models of the Disease. Progress in neurobiology. 2017;155:57–75. doi: 10.1016/j.pneurobio.2016.04.006 27107797 PMC5073045

[25] Yoritaka A, Hattori N, Uchida K, Tanaka M, Stadtman ER, Mizuno Y. Immunohistochemical Detection of 4-Hydroxynonenal Protein Adducts in Parkinson Disease. Proceedings of the National Academy of Sciences of the United States of America. 1996;93(7):2696–701. Epub 1996/04/02. doi: 10.1073/pnas.93.7.2696 ; PubMed Central PMCID: PMC39693. 8610103 PMC39693

[26] Ye Z, Li C, Liu S, Liang H, Feng J, Lin D, et al. Dl-3-n-Butylphthalide Activates Nrf2, Inhibits Ferritinophagy, and Protects MES23. 5 Dopaminergic Neurons From Ferroptosis. Chemico-biological interactions. 2023:110604. doi: 10.1016/j.cbi.2023.110604 37315914

[27] Yang W, Stockwell B. Synthetic Lethal Screening Identifies Compounds Activating Iron-Dependent, Nonapoptotic Cell Death in Oncogenic-RAS-Harboring Cancer Cells. Chem Biol. 2008;15(3):234–45. doi: 10.1016/j.chembiol.2008.02.010 18355723 PMC2683762

[28] Hinman A, Holst CR, Latham JC, Bruegger JJ, Ulas G, McCusker KP, et al. Vitamin E Hydroquinone is an Endogenous Regulator of Ferroptosis via Redox Control of 15-Lipoxygenase. PloS one. 2018;13(8):e0201369. Epub 2018/08/16. doi: 10.1371/journal.pone.0201369 ; PubMed Central PMCID: PMC6093661 30110365 PMC6093661

[29] Kahn-Kirby AH, Amagata A, Maeder CI, Mei JJ, Sideris S, Kosaka Y, et al. Targeting Ferroptosis: A Novel Therapeutic Strategy for the Treatment of Mitochondrial Disease-Related Epilepsy. PloS one. 2019;14(3):e0214250. Epub 2019/03/29. doi: 10.1371/journal.pone.0214250 ; PubMed Central PMCID: PMC6438538. 30921410 PMC6438538

[30] Pacifici M, Peruzzi F. Isolation and Culture of Rat Embryonic Neural Cells: A Quick Protocol. J Vis Exp. 2012;63. doi: e3965 10.3791/3965 PMC3466945 22664838

[31] Wan O, Chung K. The Role of Alpha-Synuclein Oligomerization and Aggregation in Cellular and Animal Models of Parkinson’s Disease. PloS one. 2012;7(6):e38545. doi: 10.1371/journal.pone.0038545 22701661 PMC3373518

[32] Saidi N, Nsibi A, Mani S, Saoud H, Messaoudi I. Unilateral 6-hydroxydopaminelesioned Rat as Relevant Model to Study the Pain Related to Parkinson’s Disease. Neurol Neurobiol. 2019;1:2613–7828.

[33] Su RJ, Zhen JL, Wang W, Zhang JL, Zheng Y, Wang XM. Time-course behavioral features are correlated with Parkinson’s disease‑associated pathology in a 6-hydroxydopamine hemiparkinsonian rat model. Molecular medicine reports. 2018;17(2):3356–63. 29257290 10.3892/mmr.2017.8277 PMC5783532

[34] Yagnik G, Liu Z, Rothschild KJ, Lim MJ. Highly Multiplexed Immunohistochemical MALDI-MS Imaging of Biomarkers in Tissues. J Am Soc Mass Spectrom. 2021;32(4):977–88. doi: 10.1021/jasms.0c00473 33631930 PMC8033562

[35] Alza N, Iglesias González P, Conde M, Uranga R, Salvador G. Lipids at the Crossroad of α-Synuclein Function and Dysfunction: Biological and Pathological Implications. Front Cell Neurosci. 2019;13:175. 31118888 10.3389/fncel.2019.00175 PMC6504812

[36] Ruipérez V, Darios F, Davletov B. Alpha-Synuclein, Lipids and Parkinson’s Disease. Prog Lipid Res. 2010;49(4):420–8. doi: 10.1016/j.plipres.2010.05.004 20580911

[37] Shamoto-Nagai M, Hisaka S, Naoi M, Maruyama W. Modification of α-Synuclein by Lipid Peroxidation Products Derived From Polyunsaturated Fatty Acids Promotes Toxic Oligomerization: Its Relevance to Parkinson Disease. J Clin Biochem Nutr. 2018;62(3):207–12. 29892158 10.3164/jcbn.18-25 PMC5990400

[38] Ugalde C, Lawson V, Finkelstein D, Hill A. The Role of Lipids in α-Synuclein Misfolding and Neurotoxicity. The Journal of biological chemistry. 2019;294(23):9016–28. 31064841 10.1074/jbc.REV119.007500 PMC6556586

[39] Chesselet M-F, Richter F, Zhu C, Magen I, Watson MB, Subramaniam SR. A progressive mouse model of Parkinson’s disease: the Thy1-aSyn (“Line 61”) mice. Neurotherapeutics: the journal of the American Society for Experimental NeuroTherapeutics. 2012;9:297–314. doi: 10.1007/s13311-012-0104-2 22350713 PMC3337020

[40] Rockenstein E, Mallory M, Hashimoto M, Song D, Shults CW, Lang I, Masliah E. Differential neuropathological alterations in transgenic mice expressing α‐synuclein from the platelet‐derived growth factor and Thy‐1 promoters. Journal of neuroscience research. 2002;68(5):568–78. 12111846 10.1002/jnr.10231

[41] Torres EM, Lane EL, Heuer A, Smith GA, Murphy E, Dunnett SB. Increased efficacy of the 6-hydroxydopamine lesion of the median forebrain bundle in small rats, by modification of the stereotaxic coordinates. J Neurosci Methods. 2011;200(1):29–35. Epub 2011/07/05. doi: 10.1016/j.jneumeth.2011.06.012 . 21723319

[42] Mahoney-Sánchez L, Bouchaoui H, Ayton S, Devos D, Duce JA, Devedjian J-C. Ferroptosis and Its Potential Role in the Physiopathology of Parkinson’s Disease. Progress in neurobiology. 2021;196:101890. Epub 2020/07/30. doi: 10.1016/j.pneurobio.2020.101890 . 32726602

[43] Kagan VE, Mao G, Qu F, Angeli JP, Doll S, Croix CS, et al. Oxidized Arachidonic and Adrenic PEs Navigate Cells to Ferroptosis. Nat Chem Biol. 2017;13(1):81–90. Epub 2016/11/15. doi: 10.1038/nchembio.2238 ; PubMed Central PMCID: PMC5506843. 27842066 PMC5506843

[44] Rai G, Joshi N, Perry S, Yasgar A, Schultz L, Jung JE, et al. Discovery of ML351, a potent and selective inhibitor of human 15-lipoxygenase-1. Probe Reports from the NIH Molecular Libraries Program [Internet]. 2014.

[45] Sadeghian H, Jabbari A. 15-Lipoxygenase inhibitors: a patent review. Expert opinion on therapeutic patents. 2016;26(1):65–88. doi: 10.1517/13543776.2016.1113259 26560362

[46] Van Leyen K, Arai K, Jin G, Kenyon V, Gerstner B, Rosenberg PA, et al. Novel Lipoxygenase Inhibitors as Neuroprotective Reagents. Journal of neuroscience research. 2008;86(4):904–9. doi: 10.1002/jnr.21543 17960827 PMC2759176

[47] Xu J, Zhang Y, Xiao Y, Ma S, Liu Q, Dang S, et al. Inhibition of 12/15-Lipoxygenase by Baicalein Induces Microglia PPARβ/δ: A Potential Therapeutic Role for CNS Autoimmune Disease. Cell death & disease. 2013;4(4):e569–e. 23559003 10.1038/cddis.2013.86 PMC3668632

[48] Costa I, Barbosa DJ, Benfeito S, Silva V, Chavarria D, Borges F, et al. Molecular mechanisms of ferroptosis and their involvement in brain diseases. Pharmacology & Therapeutics. 2023:108373. doi: 10.1016/j.pharmthera.2023.108373 36894028

[49] Dixon SJ, Lemberg KM, Lamprecht MR, Skouta R, Zaitsev EM, Gleason CE, et al. Ferroptosis: An Iron-Dependent Form of Nonapoptotic Cell Death. Cell. 2012;149(5):1060–72. Epub 2012/05/29. doi: 10.1016/j.cell.2012.03.042 ; PubMed Central PMCID: PMC3367386. 22632970 PMC3367386

[50] Xu T, Ding W, Ji X, Ao X, Liu Y, Yu W, et al. Molecular Mechanisms of Ferroptosis and Its Role in Cancer Therapy. Journal of cellular and molecular medicine. 2019;23(8):4900–12. Epub 2019/06/25. doi: 10.1111/jcmm.14511 ; PubMed Central PMCID: PMC6653007. 31232522 PMC6653007

[51] Hernandez DG, Reed X, Singleton AB. Genetics in Parkinson Disease: Mendelian Versus Non-Mendelian Inheritance. J Neurochem. 2016;139 Suppl 1(Suppl 1):59–74. Epub 2016/04/20. doi: 10.1111/jnc.13593 ; PubMed Central PMCID: PMC5155439. 27090875 PMC5155439

[52] Baba M, Nakajo S, Tu PH, Tomita T, Nakaya K, Lee VM, et al. Aggregation of Alpha-Synuclein in Lewy Bodies of Sporadic Parkinson’s Disease and Dementia With Lewy Bodies. The American Journal of Pathology. 1998;152(4):879–84. Epub 1998/04/18. ; PubMed Central PMCID: PMC1858234. 9546347 PMC1858234

[53] Wakabayashi K, Tanji K, Mori F, Takahashi H. The Lewy Body in Parkinson’s Disease: Molecules Implicated in the Formation and Degradation of Alpha-Synuclein Aggregates. Neuropathology: official journal of the Japanese Society of Neuropathology. 2007;27(5):494–506. Epub 2007/11/21. doi: 10.1111/j.1440-1789.2007.00803.x . 18018486

[54] Trojanowski JQ, Goedert M, Iwatsubo T, Lee VM. Fatal Attractions: Abnormal Protein Aggregation and Neuron Death in Parkinson’s Disease and Lewy Body Dementia. Cell Death and Differentiation. 1998;5(10):832–7. Epub 1999/04/16. doi: 10.1038/sj.cdd.4400432 . 10203692

[55] Iwatsubo T. Aggregation of Alpha-Synuclein in the Pathogenesis of Parkinson’s Disease. Journal of neurology. 2003;250 Suppl 3:Iii11–4. Epub 2003/10/28. doi: 10.1007/s00415-003-1303-x . 14579119

[56] Zhang S, Eitan E, Wu TY, Mattson MP. Intercellular Transfer of Pathogenic α-Synuclein by Extracellular Vesicles Is Induced by the Lipid Peroxidation Product 4-Hydroxynonenal. Neurobiology of aging. 2018;61:52–65. Epub 2017/10/17. doi: 10.1016/j.neurobiolaging.2017.09.016 ; PubMed Central PMCID: PMC5705257. 29035751 PMC5705257

[57] Manzanza NO, Sedlackova L, Kalaria RN. Alpha-Synuclein Post-Translational Modifications: Implications for Pathogenesis of Lewy Body Disorders. Frontiers in Aging Neuroscience. 2021;13:690293. Epub 2021/07/13. doi: 10.3389/fnagi.2021.690293 ; PubMed Central PMCID: PMC8267936. 34248606 PMC8267936

[58] Nübling GS, Levin J, Bader B, Lorenzl S, Hillmer A, Högen T, et al. Modelling Ser129 Phosphorylation Inhibits Membrane Binding of Pore-Forming Alpha-Synuclein Oligomers. PloS one. 2014;9(6):e98906. Epub 2014/06/10. doi: 10.1371/journal.pone.0098906 ; PubMed Central PMCID: PMC4049638. 24911099 PMC4049638

[59] Paillé V, Henry V, Lescaudron L, Brachet P, Damier P. Rat model of Parkinson’s disease with bilateral motor abnormalities, reversible with levodopa, and dyskinesias. Movement disorders: official journal of the Movement Disorder Society. 2007;22(4):533–9. doi: 10.1002/mds.21308 . 17230470

[60] Ferraz AC, Matheussi F, Szawka RE, Rizelio V, Delattre AM, Rigon P, et al. Evaluation of estrogen neuroprotective effect on nigrostriatal dopaminergic neurons following 6-hydroxydopamine injection into the substantia nigra pars compacta or the medial forebrain bundle. Neurochem Res. 2008;33(7):1238–46. Epub 20080209. doi: 10.1007/s11064-007-9575-7 . 18259859

[61] Tang J, Xu Y, Liu C, Fang Y, Cao S, Zhao C, et al. PET imaging with [(18)F]FP-(+)-DTBZ in 6-OHDA-induced partial and full unilaterally-lesioned model rats of Parkinson’s disease and the correlations to the biological data. Nucl Med Biol. 2020;90–91:1–9. Epub 20200815. doi: 10.1016/j.nucmedbio.2020.08.002 . 32861175

[62] Kumari N, Luthra PM. Establishment of a 6-OHDA Induced Unilaterally Lesioned Male Wistar Rat Model of Parkinson’s Disease. Methods in molecular biology (Clifton, NJ). 2024;2761:491–8. doi: 10.1007/978-1-0716-3662-6_33 . 38427257

[63] Deumens R, Blokland A, Prickaerts J. Modeling Parkinson’s disease in rats: an evaluation of 6-OHDA lesions of the nigrostriatal pathway. Experimental neurology. 2002;175(2):303–17. doi: 10.1006/exnr.2002.7891 . 12061862

[64] Devos D, Labreuche J, Rascol O, Corvol J-C, Duhamel A, Guyon Delannoy P, et al. Trial of deferiprone in Parkinson’s disease. New England Journal of Medicine. 2022;387(22):2045–55. doi: 10.1056/NEJMoa2209254 36449420

[65] Bayır H, Anthonymuthu TS, Tyurina YY, Patel SJ, Amoscato AA, Lamade AM, et al. Achieving life through death: redox biology of lipid peroxidation in ferroptosis. Cell chemical biology. 2020;27(4):387–408. doi: 10.1016/j.chembiol.2020.03.014 32275865 PMC7218794

[66] Percário S, da Silva Barbosa A, Varela ELP, Gomes ARQ, Ferreira MES, Moreira TdNA, Dolabela MF. Oxidative Stress in Parkinson’s Disease: Potential Benefits of Antioxidant Supplementation. 2020. 10.1155/2020/2360872 PMC7576349 33101584

