# Supplementary figures and images for: Targeting ferroptosis with the lipoxygenase inhibitor PTC-041 as a therapeutic strategy for the treatment of Parkinson’s disease
Source: PLoS One. 2024 Sep 18;19(9):e0309893. doi: 10.1371/journal.pone.0309893 (PMC11410249; doi:10.1371/journal.pone.0309893)

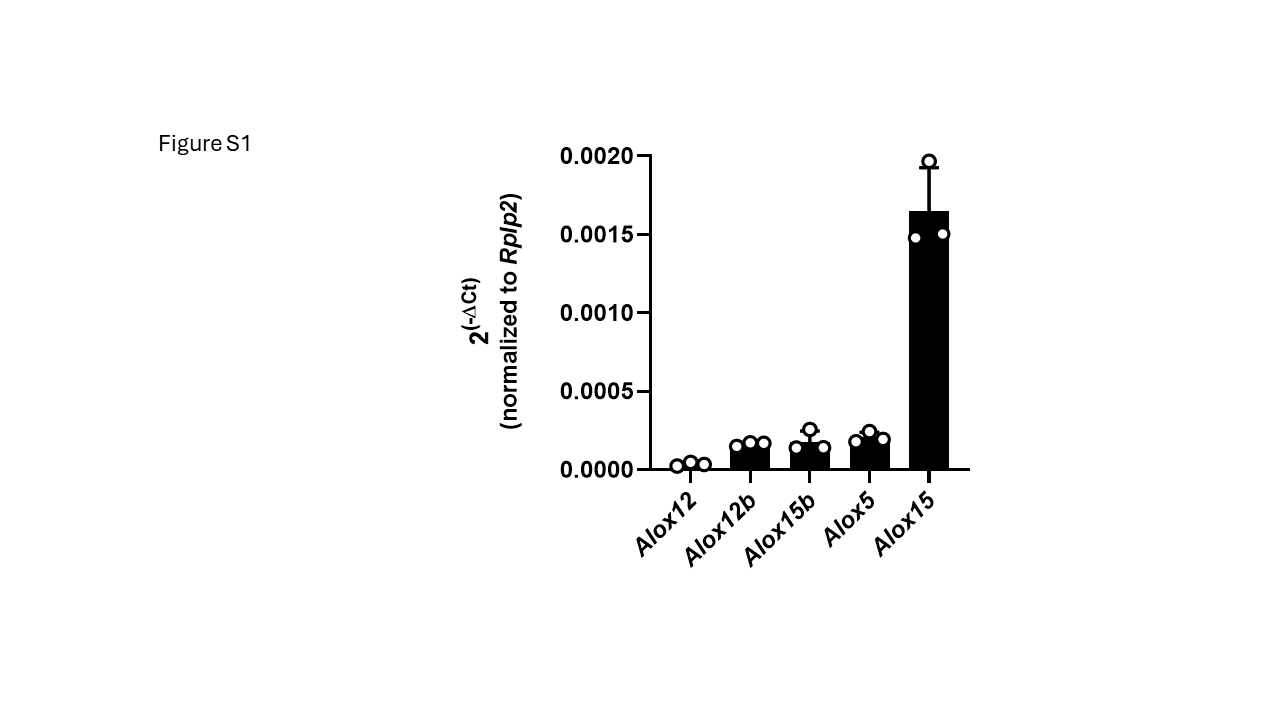

Supplement: S1 Fig — Alox gene expression levels were detected by qRT-PCR in primary rat midbrain neuron co-cultures at DIV1. All data are presented as mean ± SEM of n = 3 wells/condition, n = 1 experiment. (TIF) [file pone.0309893.s001.tif]
