# Supplementary material for: Targeting ferroptosis with the lipoxygenase inhibitor PTC-041 as a therapeutic strategy for the treatment of Parkinson’s disease
Source: PLoS One. 2024 Sep 18;19(9):e0309893. doi: 10.1371/journal.pone.0309893 (PMC11410249; doi:10.1371/journal.pone.0309893)
Supplement: S1 Table — Cell survival was assessed by CTG 2.0 assay 24 hours after RSL3 (2 μM) and concomitant compound treatment (24-point dose response). Rescue potency was determined by standard 4-parameter curve fitting (Dotmatics), with the concentration for half-maximal rescue activity (EC50) reported here. The potency of each compound was determined in 3 independent patient fibroblast cultures (ND40070, ND40078, and ND30116). The mean and SEM across the 3 donors’ cells were calculated. (DOCX) [file pone.0309893.s002.docx]

| Compound | Reported mechanism | RSL3 survival EC_50_ (nM) Mean ± SEM (n) | Fold difference in potency (versus PTC-041) |
| --- | --- | --- | --- |
| Ferrostatin-1 | 15-LO/PEPB1 complex (*63*) | 43 ± 2 (3) | 0.6 |
| **PTC-041** | **5/15-LO inhibitor** | **67 ± 6 (3)** | **1** |
| Idebenone | Mitochondrial antioxidant | 296 ± 17 (3) | 4 |
| NDGA | Pan-LO inhibitor | 390 ± 29 (3) | 6 |
| Baicalein | 12/15-LO inhibitor | 458 ± 52 (3) | 7 |
| Cannabidiol | Endocannabinoid system | 594 ± 31 (3) | 9 |
| PD146176 | 12/15-LO inhibitor | 893 ± 131 (3) | 13 |
| Zileuton | 5-LO inhibitor | 964 ± 59 (3) | 14 |
| PF-9184 | mPGES-1 inhibitor | 1628 ± 170 (3) | 24 |
| ML351 | 15-LO inhibitor | 2874 ± 178 (3) | 43 |
| Edaravone | Unknown | 7320 ± 299 (3) | 109 |
| Riluzole | Unknown | >30000 (3) | >448 |
| Ebselen | Txn system | >30000 (3) | >448 |
| CJ13610 | 5-LO inhibitor | >30000 (3) | >448 |
| Deferiprone | Iron chelator | >30000 (3) | >448 |
| Indomethacin | COX inhibitor | >30000 (3) | >448 |
| MK-886 | FLAP (5-LO) inhibitor | >30000 (3) | >448 |
| NS-938 | COX2 inhibitor | >30000 (3) | >448 |
| Licofelone | COX/LO inhibitor | >30000 (3) | >448 |
| Z-VAD-FMK | Pan-caspase inhibitor | >30000 (3) | >448 |
| PF04191834 | 5-LO inhibitor | >30000 (3) | >448 |
| Deferoxamine | Iron chelator | 33833 ± 5260 (3) | 505 |
